# Reprogramming Dysfunctional Dendritic Cells by a Versatile Catalytic Dual Oxide Antigen-Captured Nanosponge for Remotely Enhancing Lung Metastasis Immunotherapy

**DOI:** 10.1021/acsnano.4c09525

**Published:** 2024-12-31

**Authors:** Min-Ren Chiang, Chin-Wei Hsu, Wan-Chi Pan, Ngoc-Tri Tran, Yu-Sheng Lee, Wen-Hsuan Chiang, Yu-Chen Liu, Ya-Wen Chen, Shih-Hwa Chiou, Shang-Hsiu Hu

**Affiliations:** † Department of Biomedical Engineering and Environmental Sciences, National Tsing Hua University, Hsinchu 300044, Taiwan; ‡ Department of Chemical Engineering, National Chung Hsing University, Taichung 402, Taiwan; ^§^Laboratory for Human Immunology (Single Cell Genomics), WPI Immunology Frontier Research Center, ^¶^Center for Infectious Disease Education and Research (CiDER)Osaka University, Osaka 565-0871, Japan; ∥ National Institute of Cancer Research, National Health Research Institutes, Miaoli County 35053, Taiwan; ⊥ Institute of Pharmacology, College of Medicine, National Yang Ming Chiao Tung University, Hsinchu, Taipei 112304, Taiwan; # Department of Medical Research, Veterans General Hospital, Taipei, Taipei 112304, Taiwan

**Keywords:** lung metastasis, antigen capture, immunotherapy, nanozymes, T cell infiltration

## Abstract

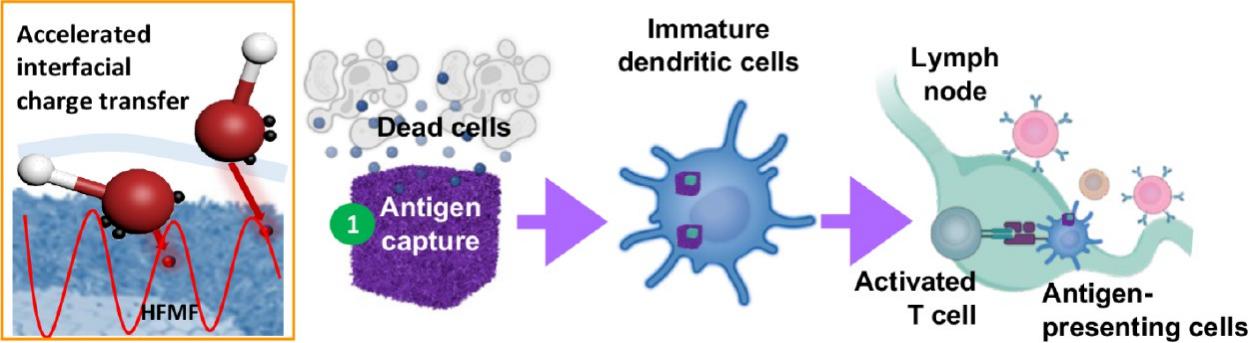

Dendritic cells (DCs) play a crucial role in initiating antitumor immune responses. However, in the tumor environment, dendritic cells often exhibit impaired antigen presentation and adopt an immunosuppressive phenotype, which hinders their function and reduces their ability to efficiently present antigens. Here, a dual catalytic oxide nanosponge (DON) doubling as a remotely boosted catalyst and an inducer of programming DCs to program immune therapy is reported. Intravenous delivery of DON enhances tumor accumulation via the marginated target. At the tumor site, DON incorporates cerium oxide nanozyme (CeO_2_)-coated iron oxide nanocubes as a peroxide mimicry in cancer cells, promoting sustained ROS generation and depleting intracellular glutathione, i.e., chemodynamic therapy (CDT). Upon high-frequency magnetic field (HFMF) irradiation, CDT accelerates the decomposition of H_2_O_2_ and the subsequent production of more reactive oxygen species, known as Kelvin’s force laws, which promote the cycle between Fe^3+^/Fe^2+^ and Ce^3+^/Ce^4+^ in a sustainable active surface. HFMF-boosted catalytic DON promotes tumors to release tumor-associated antigens, including neoantigens and damage-associated molecular patterns. Then, the porous DON acts as an antigen transporter to deliver autologous tumor-associated antigens to program DCs, resulting in sustained immune stimulation. Catalytic DON combined with the immune checkpoint inhibitor (anti-PD1) in lung metastases suppresses tumors and improves survival over 40 days.

## 1 Introduction

Lung metastasis is considered one of the most feared malignancies, with a five-year survival rate of less than 10%.^1^ Despite recent advances in lung metastasis treatments such as radiotherapy and antibody-targeted therapy, complete eradication of metastatic lung cancer remains challenging.^2−4^ Recent immunotherapy holds the promise of triggering a natural immune response to fight cancer,^5−8^ but poor vascularization in invasive clusters (usually <100 mm^3^) and low presence of tumor-associated antigens in metastases exacerbate the dilemma of diminished physical activity of T cells and cancer cells.^9,10^ Recent studies have also shown that dendritic cells (DCs) in the tumor microenvironment exhibit an immunosuppressive metabolic state.^11−13^ Specifically, changes and reductions in antigen signaling combined with regulatory dendritic cells (DCs) affect the immune response, impairing the priming of cytotoxic T lymphocytes. Thus, limited immunogenicity and autoimmune side effects hinder the efficacy of immune checkpoint blockers, such as anti-PD1, with serious consequences.^14−16^

To address these challenges, activation of DCs to induce tumor-specific targeting cytotoxic T lymphocytes exhibits a promising strategy to maximize the benefits of immunotherapy. Activation of cytotoxic T lymphocytes relies on APCs, in which immunogenicity is enhanced through the application of immunogenic substances.^17−21^ In this regard, various delivery systems are able to transport antigens to DCs and lymph nodes to establish antigen-specific interactions with T cells. In the past, tumor cell membranes, tumor lysates, or tumor cell exosomes have been used to activate epitopes produced by tumors.^22−25^ However, this approach requires complex procedures and preparation, and the delivered antigens often fail to elicit effective immune responses.^26−30^

To initiate DC responses, another approach involves the eradication of cancer cells to induce the production of tumor-associated antigens (TAA). Within this context, reactive oxygen species (ROS), pivotal in cellular signaling, are oxygen molecules subjected to derivatization, particularly hydrogen peroxide (H_2_O_2_), exhibiting excessive expression and capable of inflicting potent oxidative damage within cancer cells.^26,27^ These ROS responders enable cancer therapeutic redox by promoting intracellular conversion of H_2_O_2_ to ·OH.^28−30^ This process triggers a Fenton-like reaction within the tumor milieu, wherein H_2_O_2_ undergoes disproportionation to generate toxic ·OH, thereby facilitating the oxidation and impairment of intracellular proteins and organelles.^31^ Consequently, the failure of proteins and organelles can trigger genotoxic reactions and metabolic deficiencies, prompting the activation of autophagy, a self-defense mechanism.^32−34^ Within this mechanism, cells sequester cytoplasmic organelles in autophagosomes and transport them to lysosomes for degradation, aiding in the clearance of ·OH-damaged proteins and organelles for detoxification.^35,36^ Furthermore, drug resistance, particularly due to glutathione (GSH) overexpression in cancer cells, remains a significant obstacle.^24,25^ Therefore, reducing the activation of autophagy and GSH levels is crucial to enhancing the effectiveness of CDT drugs.

To attenuate autophagy activation within tumors, various metal–organic frameworks (MOFs) combined with magnetic, light, or sound stimulation were developed to effectively catalyze the conversion of hydrogen peroxide into hydroxyl radicals, surpassing the efficacy of the traditional Fenton reaction.^37−42^ The photoresponsive materials containing iron (Fe^2+^ and Fe^3+^) proceed by generating reactive oxygen species (ROS) through the photoreduction of Fe^3+^. Upon light irradiation, Fe^2+^-loaded lanthanide-doped porous particles induce local ·OH radical formation within cancer mitochondria, leading to significant mitochondrial DNA damage.^38^ Recently, remotely magneto-thermodynamic (MTD) therapy, by combining intense heat and ROS-related immunologic effects, can also overcome the obstacle of limited CDT efficacy.^39,40^ In chemistry, chloroquine is a classic autophagy inhibitor that can also block autophagy flow.^43^ Innate immunity amplifies cytokine production through activated receptors, playing a role in downstream autophagy regulation.^44−46^

**Figure 1:**
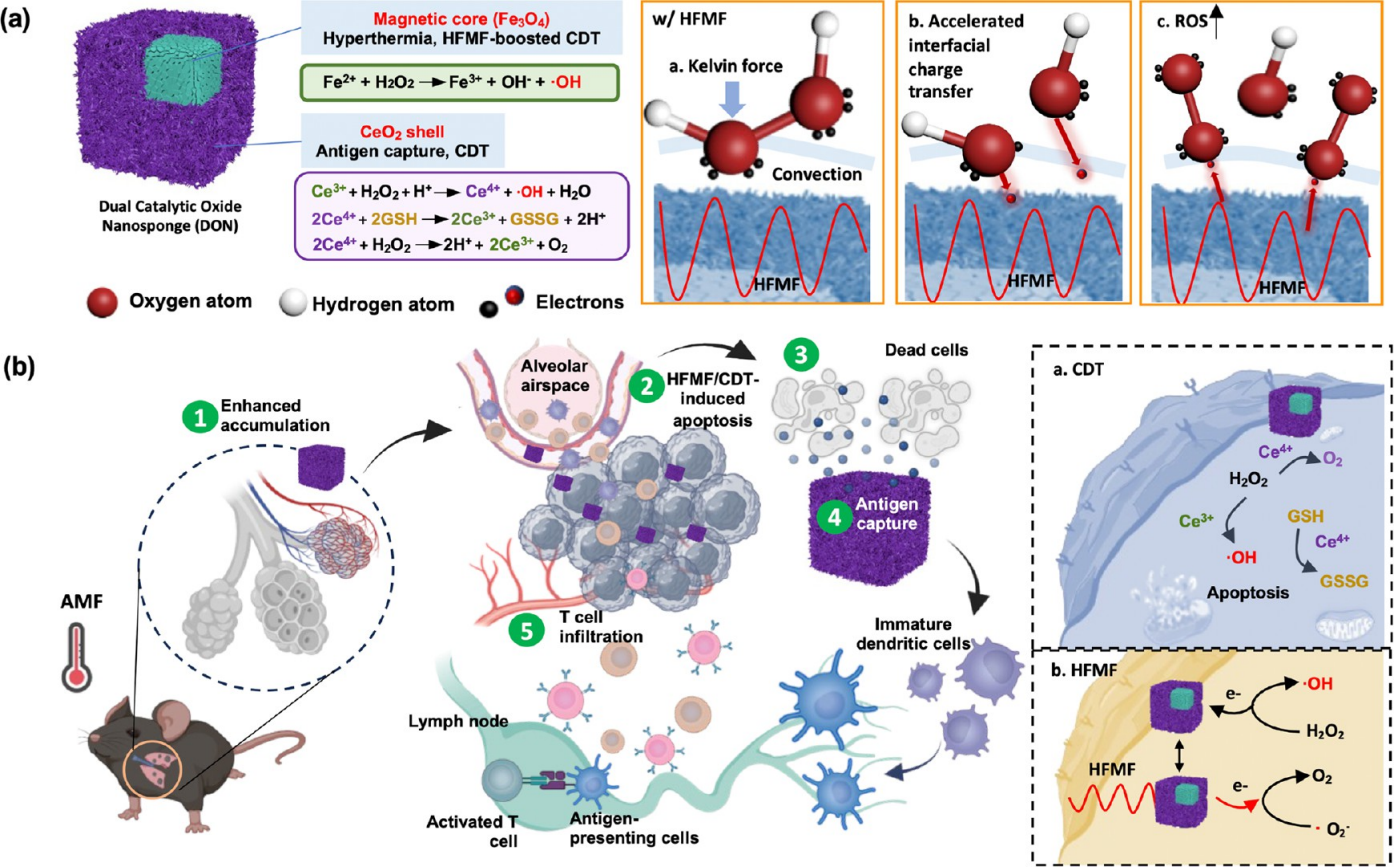
Schematic illustration of dual catalytic oxide nanosponge (DON) a dual catalyst and an inducer of T cell infiltration to program immune therapy. (a) DON consisting of cerium oxide nanozyme (CeO_2_), iron based-MOF, and iron oxide nanocubes (IO) as agents to promote the cycle between Fe^3+^/Fe^2+^ and Ce^3^+/Ce^4+^ as well as to decompose H_2_O_2_ upon a high-frequency magnetic fields (HFMF) irradiation. (b) The hyperthermia and chemodynamic therapy (CDT) via redox reactions promoted cancer cell apoptosis and release TAAs. Porous DON enhances the retention of antigen release, facilitating sustained immune stimulation and inhibiting tumor metastasis.

In immunotherapy, it relies on capturing and delivering the antigens to the lymph nodes.^47,48^ This process triggers endogenous danger signals and TAA, which in turn activate antigen-presenting cells (APCs).^46^ However, off-target delivery usually limits immunogenic cell death, and unmet expression constraints remain without prolonged antigen retention and delivery. Here, dual catalytic oxide nanosponges (DON) consisting of cerium oxide nanozyme (CeO_2_), iron-based MOF, and iron oxide nanocubes (IO) were developed for programming immune therapy and mediating DCs (Figure 1a).^49,50^ Following accumulation in the lung via the margination effect, DON permeates lung metastases by being taken up by alveolar luminal endothelial cells and leukocytes (Figure 1b). At the tumor site, a high-frequency magnetic field (HFMF) with chemodynamic therapy (CDT) accelerates charge transfer to decompose H_2_O_2_ and the subsequent production of more reactive oxygen species. This process further enhances cycling between Fe^3+^/Fe^2+^ and Ce^3+^/Ce^4+^ with impressive recyclability to facilitate the inhibition of autophagy, leading to cancer cell apoptosis and triggering the release of tumor-associated antigens (TAAs).^51−53^ Subsequently, the porous properties of DON enable the capture of these TAAs, serving as an antigen reservoir that induces immunogenic cell death. This mechanism not only ensures sustained immune stimulation but also effectively suppresses tumor metastasis at the tumor site. Moreover, the captured antigens attract additional DCs, thereby amplifying the immune response mediated by CD4^+^ and CD8^+^ T cells. Consequently, the proposed antigen capture mechanism holds significant promise for enhancing cancer immunotherapy.

## 2 Results

### 2.1 Synthesis and Characterization of PB, PC, PCA, and DON

**Figure 2:**
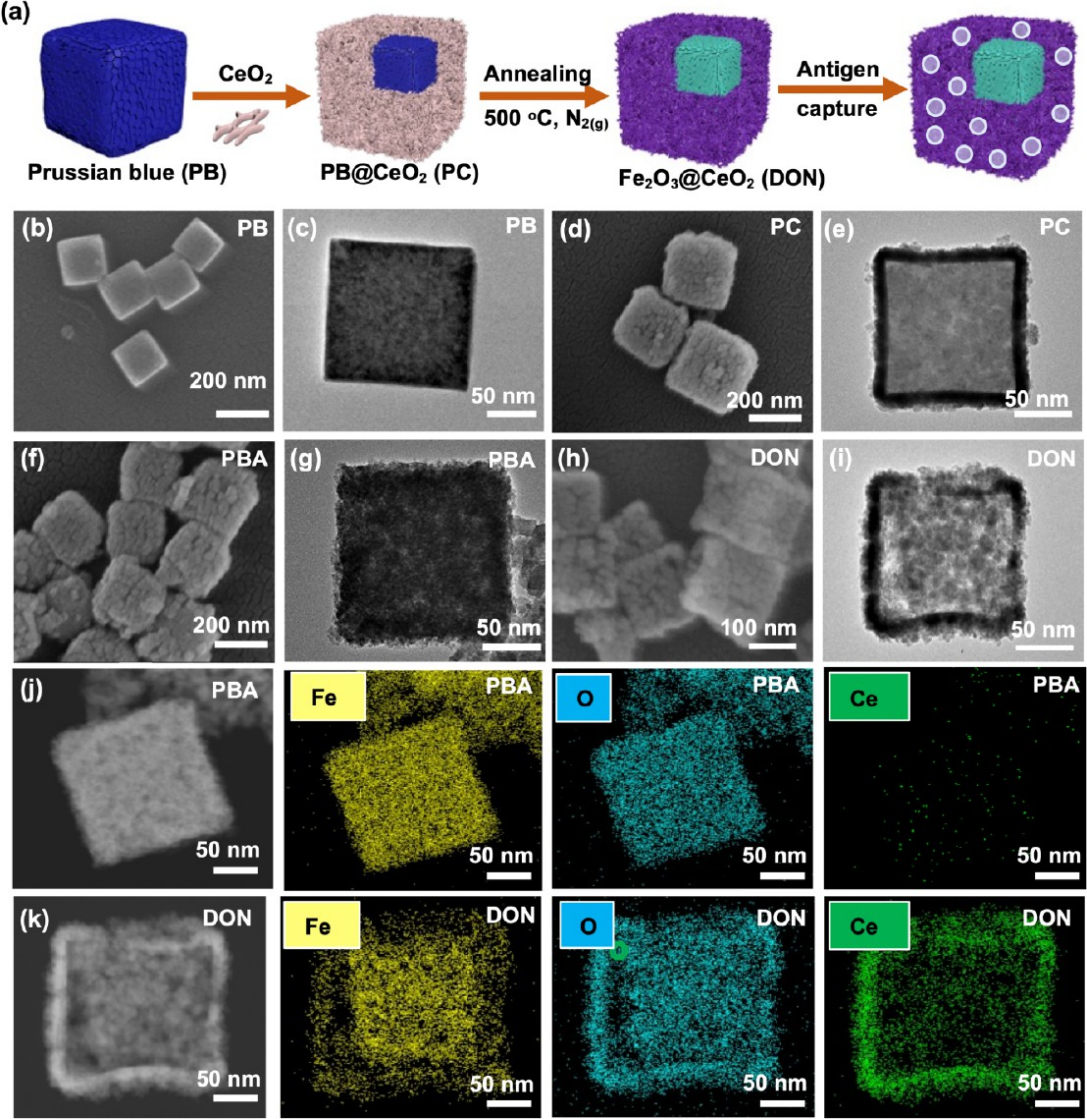
Synthesis and characterizations of PB, PC, and DON. (a) Schematic representation showing the synthesis of DON for HFMF-enhanced catalytic therapy. SEM and TEM images of (b,c) PB, (d,e) PC, (f,g) PBA, and (h,i) DON. (j,k) Element mapping analysis of PBA and DON.

The schematic diagram in Figure 2a illustrates the synthetic route to produce DON. Initially, PB was synthesized via a hydrothermal method using PVP and potassium ferricyanide as iron sources. Subsequently, a cerium(III) nitrate coating was applied on PB (Fe_4_[Fe(CN)_6_]_3_), followed by a calcination process at 450 °C, which facilitated the conversion of iron into Fe_2_O_3_.^54^ The cerium oxide coating enhances the Fenton oxidation reaction by promoting oxygen vacancies and promoting electron transfer between Fe^3+^/Fe^2+^ and Ce^3+^/Ce^4+^ ions.^55,56^ The morphologies of PB, PC, PBA, and DON were meticulously examined using scanning electron microscopy (SEM) and transmission electron microscopy (TEM) in Figure 2b-i. The findings revealed consistent size and shape across all nanocubes, which were unaffected by subsequent surface modifications or calcination. Notably, the cerium oxide coating marginally increased the size of PB from 180 to 200 nm. Moreover, the annealing process induced a coarse surface texture on the nanocubes, characteristic of mesoporous materials, potentially enhancing their antigen-capturing capabilities. To evaluate the effects of surface textures on antigen-capturing capabilities, PC and DON were used to adsorb the model protein bovine serum albumin (BSA). The results demonstrated that DON, which has a coarse surface texture, exhibited a higher efficacy in capturing BSA (Figure S1). This enhanced capturing ability is likely due to the increased surface area and the presence of carbonized surfaces, which provide more active sites for protein adsorption and stronger interactions between the protein molecules and the nanoparticle surface. The results and discussion are added in the Supporting Information and the article.

TEM analysis depicted CeO_2_ tending to grow on the surface of PB, forming a core–shell structure rather than independently forming distinct particles. This phenomenon can be elucidated by the hydrolysis of (CH_2_)_6_N_4_, leading to a decrease in water concentration within the ethanol/water solution, thereby resulting in a low nucleation and growth rate of cerium oxide (eqs 1 and 2). Additionally, under alkaline conditions, Fe(III) ions in Fe_4_[Fe(CN)_6_]_3_ interact with hydroxide ions to produce insoluble Fe(OH)_3_, creating vacancy sites for Ce^3+^ deposition on the PB.^57,58^ Element mapping results corroborated the presence of cerium oxide on DON but not on PBA (Figure 2j-k).
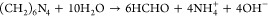
1
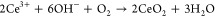
2

### 2.2 Physicochemical Characterization of PB, PC, PBA, and DON

**Figure 3:**
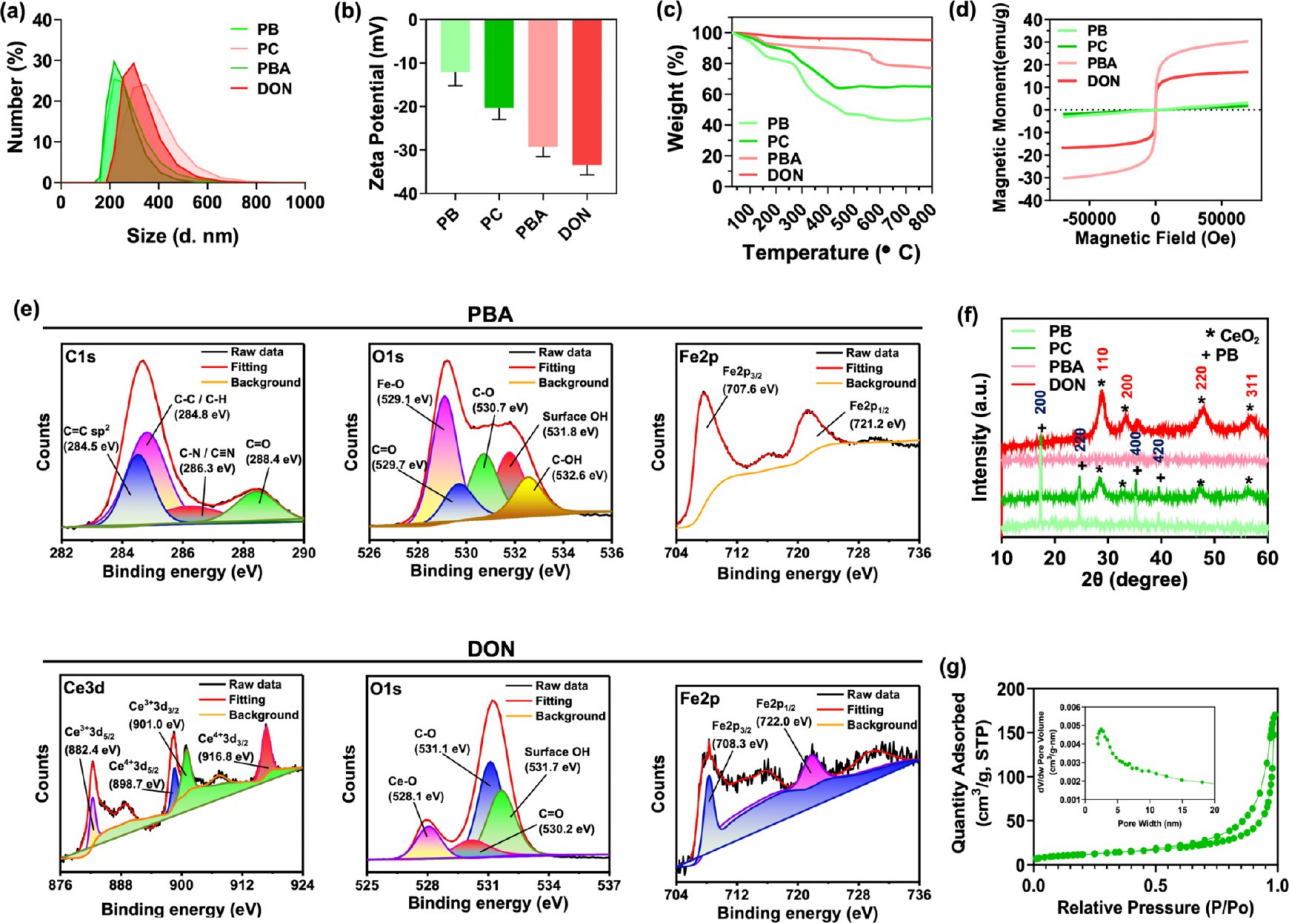
Physicochemical characterization of PB, PC, PBA, and DON. (a) Size distributions and (b) surface charges of PB, PC, PBA, and DON. (c) Thermogravimetric analysis curves and (d) field-dependent magnetization curves of PB, PC, PBA, and DON. (e) XPS spectrum of C 1s, O 1s, and Fe 2p of PBA and DON. (f) XRD spectrum of PB, PC, PBA, and DON. (g) BET analysis of N_2_ adsorption–desorption isotherms of DON.

The characterization of PB, PC, PBA, and DON was conducted through dynamic light scattering (DLS), confirming particle size consistency with SEM and TEM data (Figure 3a). Zeta potential measurements revealed a negative charge across all four nanocubes (Figure 3b). Thermogravimetric analysis (TGA) highlighted PB as the major contributor to weight loss, while cerium oxide-coated nanocubes exhibited enhanced thermal stability (Figure 3c). Specifically, PB exhibited 55% weight loss, whereas PC showed only 36% weight loss.

The magnetic properties of the nanocubes were assessed via magnetic hysteresis analysis using a superconducting quantum interference device magnetometer (SQUID) to evaluate postannealing outcomes (Figure 3d). The results confirmed the successful calcination of both PBA and DON. Both nanocubes displayed magnetic hysteresis, indicating their superparamagnetic nature. The saturation magnetization of PBA and DON was measured at 29.8 and 16.7 emu/g, respectively, with the reduction in magnetic moment attributed to the presence of the cerium oxide coating.

X-ray photoelectron spectroscopy (XPS) was employed to elucidate the organic/inorganic ratios and bonding in PB, PC, PBA, and DON. The C 1s spectrum in PB exhibited binding energy peaks at 284.6 and 285.4 eV, corresponding to C–C and C≡N bonds. The N 1s spectrum revealed major binding energy peaks at 397.3 and 399.3 eV, indicating Fe^2+^-(C≡N) X and Fe^3+^-(C≡N) X bonds. The O 1s spectrum showed a binding energy peak of the C–O bond at 531.2 eV. Additionally, the Fe 2p spectrum displayed two main binding energy peaks at 708.1 and 720.9 eV, corresponding to Fe 2p3/2 and Fe 2p1/2, providing evidence for the successful synthesis of PB (Figure S2a).^60−62^ In the XPS spectrum of PC, the O 1s spectrum exhibited binding energy peaks for the C–O bond at 531.2 eV and the Ce–O bond at 528 eV. Four main binding energy peaks in the Ce 3d spectra at 882.5, 898.3, 900.9, and 916.7 eV corresponded to Ce^3+^ 3d5/2, Ce^4+^ 3d5/2, Ce^3+^ 3d3/2, and Ce^4+^ 3d3/2, respectively (Figure S2b). In the survey spectra of PBA, the C 1s spectrum revealed binding energy peaks at 283.6 and 287 eV corresponding to C–C and C–O bonds. The O 1s spectrum exhibited a binding energy peak of the Fe–O bond at 529.9 eV, and the Fe 2p spectrum displayed two main binding energy peaks at 710 and 723.4 eV, corresponding to Fe 2p3/2 and Fe 2p1/2 (Figure 3e).^63,64^ For XPS spectra of DON, the Fe binding energy peak was inconspicuous due to the surface coating of cerium oxide. The O 1s spectrum revealed binding energy peaks at 528 and 531.2 eV, corresponding to Ce–O and C–O bonds. The Fe 2p spectrum exhibited two main binding energy peaks at 710 and 723.4 eV. Four main binding energy peaks in the Ce 3d spectra at 882.5, 898.3, 900.9, and 916.7 eV corresponded to Ce^3+^ 3d5/2, Ce^4+^ 3d5/2, Ce^3+^ 3d3/2, and Ce^4+^ 3d3/2.

To further characterize the nanocube’s crystallization and evaluate the cerium oxide coating, X-ray diffraction (XRD) analysis was performed as shown in Figure 3f. The diffraction peaks at 17.3°, 24.5°, 35.1°, and 39.5° corresponded to the standard markers of PB, representing crystal planes (200), (220), (400), and (420), respectively, indicative of a face-centered cubic lattice in the synthesized PB nanocubes.^59^ The XRD results for DON displayed peaks at 28.5°, 33.5°, 47.6°, and 56.8°, corresponding to crystal planes (110), (200), (220), and (311), affirming the cubic fluorite structure of the cerium oxide.^60^ The annealing process retained the cerium oxide on the PB surface. Moreover, nitrogen adsorption–desorption isotherms, analyzed using the Brunauer–Emmett–Teller (BET) method, were employed to assess the porous structure of PB, PC, PBA, and DON in Figures 3g and S3. The results revealed that the surface area of PB was 248 m^2^/g, with no apparent porous structure indicated in the pore volume plot. For PC, the surface area was 78 m^2^/g, and the pore volume plot displayed mesoporous properties, evidenced by the peak between 2 and 5 nm. Following annealing of PB, the surface area of PBA decreased to 23 m^2^/g, and mesoporous pores were evident, albeit with a less pronounced peak between 5 and 7 nm. Subsequently, the surface area of DON was 42 m^2^/g, with a distinct peak between 2 and 10 nm, signifying mesoporous characteristics. We proposed that the decrease in surface area could be attributed to the structural collapse of the nanocubes or the presence of additional materials coated on the surface.

### 2.3 HFMF-Enhanced Catalytic Effects of DON

**Figure 4:**
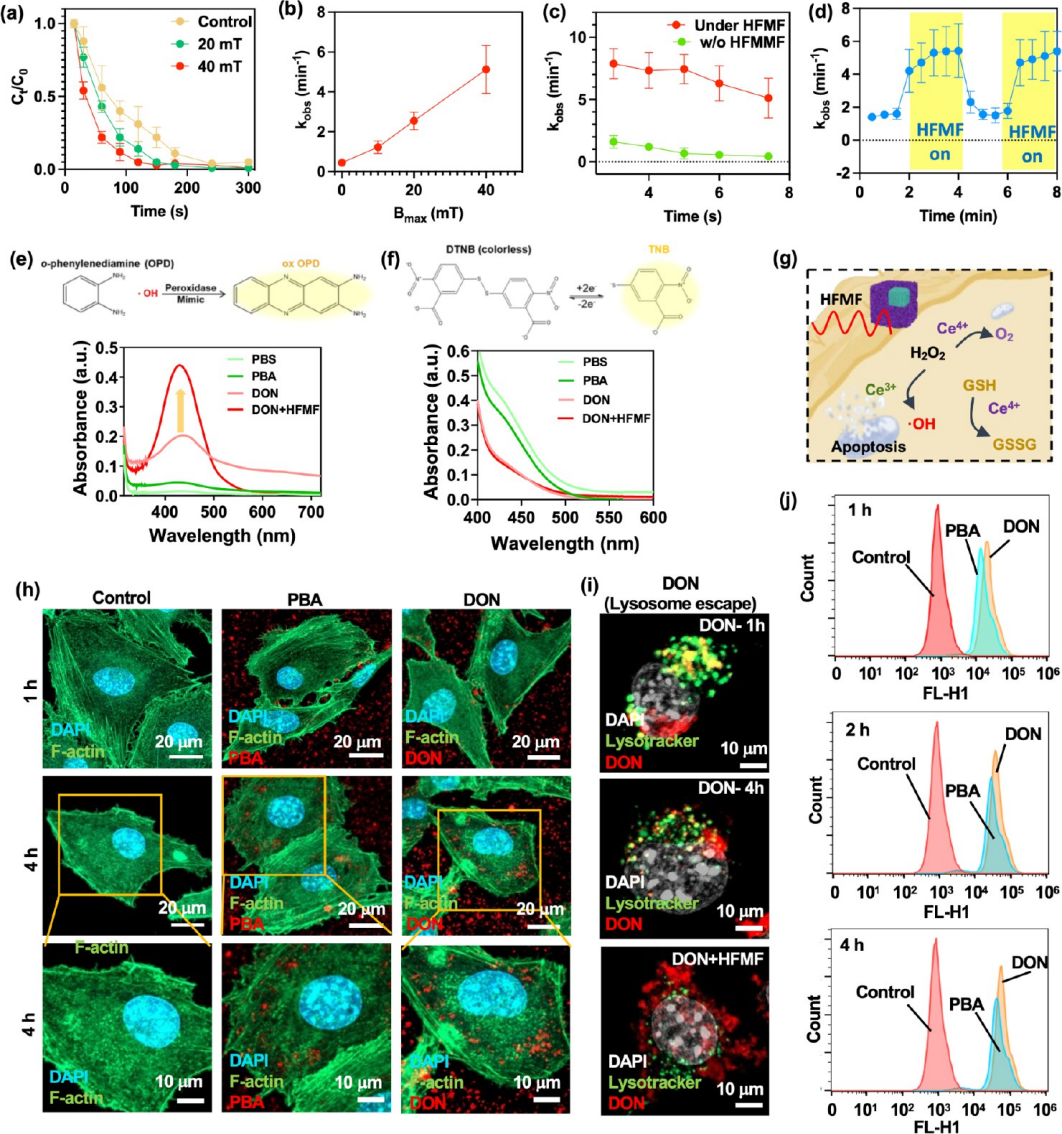
(a) Degradation patterns of rhodamine B (RhB) under an HFMF with the magnetic strengths of 0, 20, and 40 mT. RhB concentration: 10 mg/L; H_2_O_2_ concentration: 5 mM; pH: 7.4. Statistical significance was assessed using one-way ANOVA. Data represent mean ± SEM, *n* = 5. (b) Relationship between magnetic strength and reaction kinetic rate. Statistical significance was assessed using one-way ANOVA. Data represent mean ± SEM, *n* = 5. (c) Comparing the observed reaction kinetic rates (kobs) in the presence and absence of an HFMF across different pH values, while maintaining a constant dosage of 5 mL of H_2_O_2_. Statistical significance was assessed using one-way ANOVA. Data represent mean ± SEM, *n* = 5. (d) Monitoring the change in reaction kinetic rate upon activation and deactivation of the HFMF. Statistical significance was assessed using one-way ANOVA. Data represent mean ± SEM, *n* = 6. (e) The peroxidase mimic catalytic activity of PBA and DON. UV–vis absorption spectra of catalyzed oxidation of OPD (ox OPD) in an acid environment. (f) The GSH depletion of PBA and DON. (g) Mechanism of peroxidase mimic catalytic process and GSH depletion test of nanoparticles. (h) CLSM images of B16F10 cells incubated with PBA and DON. Blue, green and red represents nucleus stained with DAPI, cytoskeleton with F-Actin, and particles stained with QD, respectively. (i) CLSM images of B16F10 cells incubated with DON to evaluate the lysosomal escape effect of DON after HFMF irradiation. (j) Flow cytometry analysis of PBA and DON.

The catalytic activity of DON under an HFMF was studied by observing the degradation rate of the RhB dye in Figure 4a. Applying an external HFMF significantly accelerates the degradation rate, with complete removal of RhB achieved in 100 s under an HFMF of 40 mT. This is almost three times faster compared to the 240 s required without an HFMF. The kinetic rate constant (*k*_obs_) in this scenario is remarkably high at 5.4 min^–1^, which is 3.9 times greater than the 1.7 min^–1^ observed without an HFMF. Additionally, Figure 4b shows that *k*_obs_ increases proportionally with the magnetic field strength (*B*_max_) of the HFMF, underscoring the critical role of the magnetic field in enhancing the degradation rate. Further investigations into the degradation of RhB dye across various pH levels, with and without HFMF, are shown in Figure 4c. The results indicate that *k*_obs_ remains elevated across a broad pH range (from strong acidity to near neutral) when an HFMF is applied compared to cases without an HFMF. This wide pH tolerance is notably different from previously reported heterogeneous Fenton catalysts, which typically perform well only in highly acidic conditions.

To eliminate the possibility of temperature effects influencing the results, additional degradation tests were conducted using HFMF switch-on and switch-off experiments. These tests allowed for the immediate cessation of HFMF-induced factors (such as force, electron transfer, diffusion, and hydrodynamics) without a rapid decrease in temperature upon switching off the HFMF. For these experiments, a high concentration of RhB (100 mg L^–1^) was used to intentionally decelerate the otherwise ultrafast catalytic reaction. Figure 4d illustrates that the *k*_obs_ value rises quickly when the HFMF is on but drops immediately once the HFMF is turned off. This indicates that the enhanced degradation rate of the DON catalyst is primarily due to HFMF rather than an increase in temperature. This finding contrasts with previous studies that often attribute the enhanced activity to HFMF-induced heating effects.

### 2.4 Redox Imbalance and GSH Depletion of DON

The ROS generation ability of DON was assessed through the o-phenylenediamine (OPD) assay, wherein the determination of ROS levels relied on color change and the absorption peak at 417 nm of the oxidized OPD (oxOPD) on the UV–vis spectrum.^65^ The results revealed that DON, particularly when subjected to HFMF, exhibited the highest ROS values (Figure 4e). This outcome underscores the synergistic effect of cerium oxide and HFMF in enhancing the ROS production by PB, emphasizing its potential for effective tumor elimination. According to Kelvin’s force laws, the higher the magnetic field applied, the more Kelvin force was generated, and thus the faster the mass transfer. In this case, the group with HFMF has a higher oxOPD level because the interfacial electron charge transfer between the DON and H_2_O_2_ under the influence of HFMF is more beneficial for the decomposition of H_2_O_2_ and the generation of hydroxyl radicals.^65^

Similarly, in the 5,5′-dithio-bis(2-nitrobenzoic acid) (DTNB) assay, a consistent trend was observed in GSH depletion. The Fenton-like reaction induced by cerium oxide led to the consumption of GSH through the catalysis of tetravalent cerium ions (Ce^4+^). Consequently, a significant decrease in the 412 nm absorption in the UV–vis peak was noted in the DON groups in Figure 4f,g. The results from both ROS generation and GSH depletion assays collectively underscore the robust capability of DON in inducing oxidative stress and mitigating GSH-associated drug resistance mechanisms.

### 2.5 *In Vitro* Cellular Uptake of DON

To assess the B16F10 cellular uptake (a murine melanoma cell line) of PBA and DON, time-dependent analysis was conducted using confocal imaging, as shown in Figure 4h. The results indicate enhanced uptake with time increase for both PBA and DON. With modification, nanoparticles within a specific size range can passively enter cells through mechanisms like endocytosis or direct diffusion, a phenomenon termed passive cellular uptake. Nanoparticles below 200 nm, in particular, can traverse cellular membranes more readily, enhancing their cellular uptake. Moreover, unmodified nanoparticles may possess surface properties that facilitate their uptake by cells. Surface characteristics of nanoparticles can induce cellular responses, prompting their internalization. Additionally, nanoparticles may interact with cell surfaces via electrostatic forces, further facilitating their uptake. This interaction is governed by both the charge of the nanoparticles and the charge distribution on the cell membrane. The fact that DON is slightly more negative than PBA plays a contributing role in the high cell uptake through endocytosis, as most anionic nanoparticles would be internalized by caveolae-mediated pathways.^66,67^ Furthermore, the lysosomal escape effect was assessed by incubating DON with the B16 cell line (Figure 4i). The results showed that the colocalization of DON and lysosomes was disrupted after HFMF treatment, suggesting that external stimulation can induce the escape of DON from the lysosomes. Figure 4j presents the flow cytometry findings regarding cell uptake, aligning consistently with the results obtained from confocal laser scanning microscopy (CLSM) imaging.

The cell uptake and lysosome escape experiments were performed using DC2.4 cells. As shown in Figure S4a, the results demonstrated a time-dependent increase in uptake for both PBA and DON. Nanoparticles can passively enter cells via mechanisms such as endocytosis or direct diffusion, a process known as passive cellular uptake. Furthermore, the lysosomal escape effect was evaluated by incubating DON with DC2.4 cells (Figure S4b). Similar to observations in B16 cells, the colocalization of DON and lysosomes in DC2.4 cells was disrupted after HFMF treatment. This indicates that external stimulation can promote the escape of DON from lysosomes.

### 2.6 *In Vitro* Cytotoxicity and Intracellular ROS of DON

**Figure 5:**
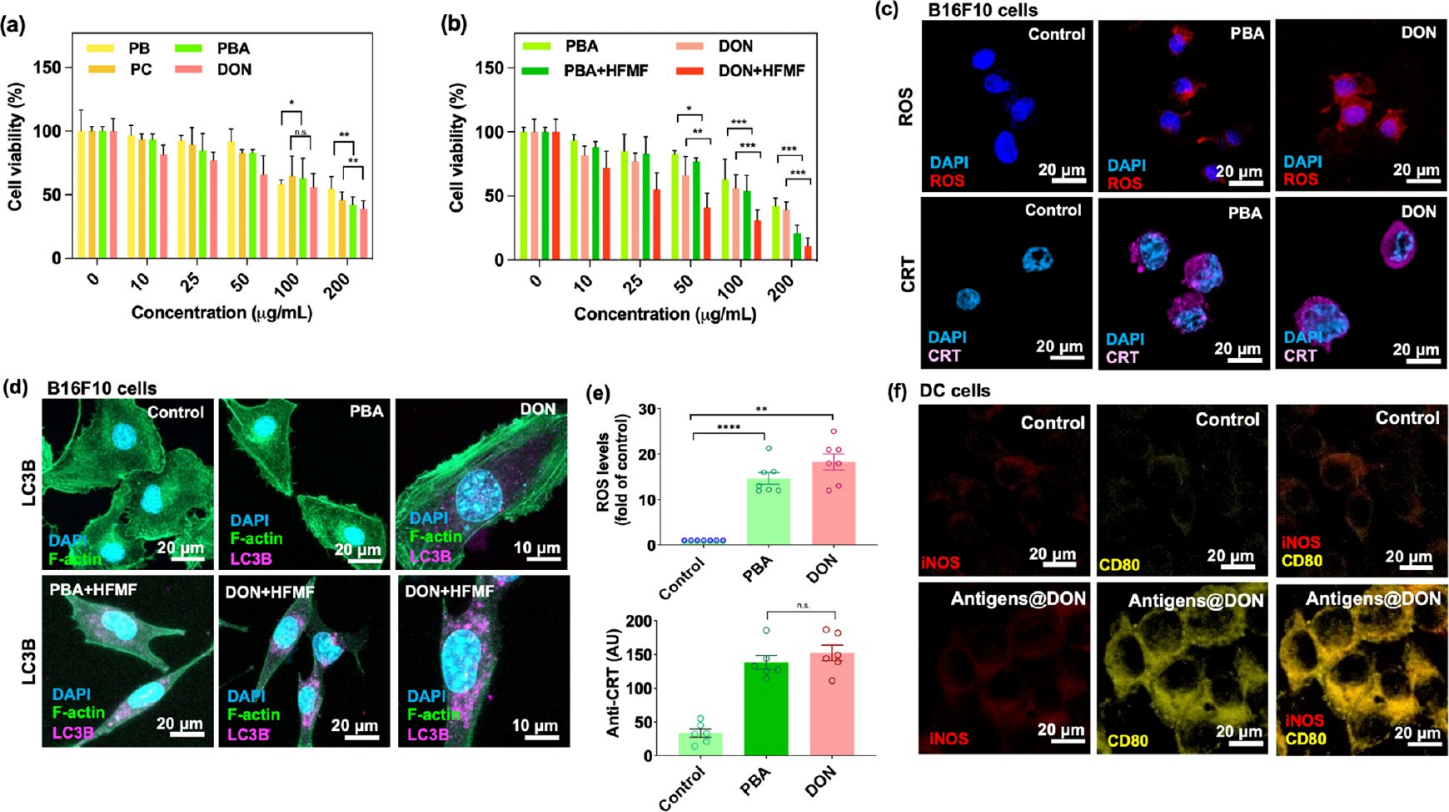
(a,b) Cell viability of B16F10 treated with PB, PC, PBA, and DON at various concentrations with and without subjecting to HFMF. Statistical significance was assessed using one-way ANOVA. Data represent mean ± SEM, *n* = 6. *p < 0.05, **p < 0.01, ***p < 0.005. (c) CLSM images of *in vitro* assessments of the intracellular hydroxyl radical ROS generation and anti-CRT via the catalysis of PBA and DON nanoparticles. (d) CLSM images of autophagy activation through the LC3B protein expression of B16F10 cells incubated with PBA, DON, PBA+HFMF, and DON+HFMF. Purple fluorescence indicates LC3B expression, serving as a measure of autophagosome abundance. (e) Quantitative levels of ROS and anti-CRT are expressed as the percentage of hydroxyl identified in control cells. Statistical significance was assessed using one-way ANOVA. Data represent mean ± SEM, *n* = 6. **p < 0.01, ****p < 0.001. (f) CLSM images of DC 2.4 cells treated with antigen@DON for 24 h.

Subsequent evaluation of cytotoxicity in B16F10 melanoma cells revealed a dose-dependent response for both PBA and DON, as shown in Figure 5a. DON exhibited higher toxicity compared to non cerium-coated PBA. This heightened cytotoxicity is attributed to the Fenton-like reaction induced by cerium oxide, consistent with prior findings on GSH depletion and ROS generation. Furthermore, in Figure 5b, the results of culturing cells with particles for 24 h followed by treatment with or without HFMF (operating at a 3.2 kW power and 50 kHz frequency) for 5 min are shown. Notably, DON exhibited more obvious cytotoxic effects compared with PBA, indicating the enhancement of the effects of chemodynamic therapy (CDT) when combined with HFMF therapy. This might be attributed to the charge transfer known as Kelvin force upon HFMF irradiation. This phenomenon significantly promotes the decomposition of H_2_O_2_, leading to an increased production of more reactive oxygen species. Furthermore, human umbilical vein endothelial cells (HUVEC) and mouse fibroblast cells (L929) were used to evaluate the toxicity of the materials. Compared to cancer cell lines, the cytotoxicity observed in HUVEC and L929 cells was lower (Figure S5). This reduced toxicity in normal cells could be attributed to their lower levels of hydrogen peroxide, resulting in reduced catalytic effects compared to cancer cells.

After evaluating the Fenton reaction by o-phenylenediamine assay and the cellular uptake ability of DON, the intracellular ROS generation capability in the cancer cell line was assessed in Figure 5c. The results demonstrated that both PBA and DON efficiently triggered the Fenton reaction, leading to ROS generation inside the cells. Furthermore, the cerium oxide coating in DON contributed to an additional Fenton-like reaction, resulting in higher ROS levels compared to the PBA group. Both PB and CeO_2_ demonstrated multifaceted enzyme-mimetic activities, encompassing catalase (CAT)-like, peroxidase (POD)-like, superoxide dismutase (SOD)-like, and glutathione peroxidase (GPx)-like functionalities. Within the acidic microenvironment characteristic of tumors, cerium oxide exhibited a predilection for functioning as POD-like, facilitating the oxidation of H_2_O_2_ into hydroxyl radicals. Additionally, in conjunction with the PB, a synergistic effect was observed, leading to an augmentation of ROS production.

Calreticulin (CRT) is a key marker of immunogenic cell death (ICD), a process initiated by the translocation of CRT from the lumen of the endoplasmic reticulum (ER) to the nuclear surface. These exposed CRT molecules serve as signals for recognition by CD91-expressing cells such as macrophages and DCs, thereby stimulating DC recruitment and promoting antigen presentation. As shown in Figure 5c, the presence of CRT (purple fluorescence) was observed in both the PBA and the DON groups. Furthermore, HMGB1 (High Mobility Group Box 1) plays a significant role in the immune response, primarily as a pro-inflammatory mediator. HMGB1 is released by cells during stress, injury, or death (especially during necrosis). When released extracellularly, it acts as a DAMP, signaling the immune system that there is damage or infection. To evaluate HMGB1 expression following particle treatments, cancer cells treated with PBA and DON showed strong HMGB1 expression (Figure S6). Upon activation, HMGB1 can be actively secreted by immune cells or passively released from damaged or stressed cells into the extracellular space, where it functions as a danger signal or DAMP. The results and discussion are added in the article and the Supporting Information. The CLSM images in Figure 5d show a strong fluorescence expression of LC3B (purple fluorescence) in PBA and DON groups. This observation highlights that all three substances are potent inducers of ICD, showing their ability to elicit this immunogenic response. The relative quantifications are also presented in Figure 5e.

To preliminarily evaluate the effects of DON on DC maturation, B16F10 cells were treated with DON+HFMF, and their released antigens were collected. These antigens, in combination with DON (antigens@DON), were then cocultured with DC2.4 cells (a murine dendritic cell line commonly used in research to study dendritic cell biology) for 24 h. Following the coculture period, the DC2.4 cells were fixed and stained for iNOS and CD80, markers used to distinguish between untreated (immature) and treated (mature) DC2.4 cells. As shown in Figure 5f, the CLSM images of DC2.4 cells treated with antigens@DON exhibited signs of maturation, indicating that the antigen-containing particles can induce DC maturation. The flow cytometry results presented in Figure S7 are consistent with the CLSM images.

### 2.7 *In Vivo* Tumor Accumulation and Biodistribution of DON

**Figure 6:**
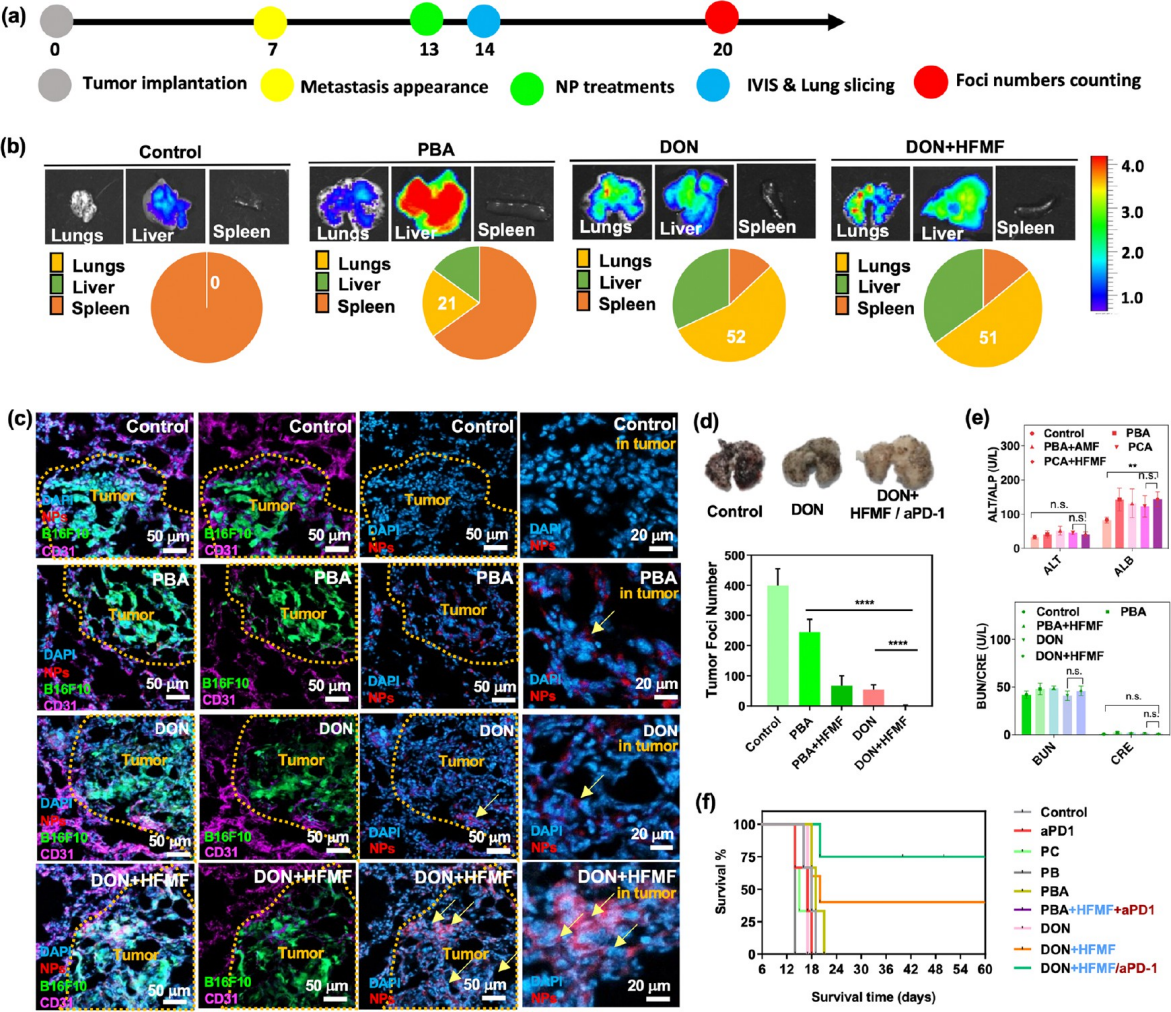
*In vivo* study. (a) Animal treatment schedule. (b) *In vivo* IVIS organ biodistribution images of control, PBA, DON, and DON+HFMF-treated mice at 24 h post-treatment. (c) CLSM images of mice bearing GFP-B16F10 lung metastases after treatment with PBA, DON, and DON+HFMF, respectively, after 24 h of treatment. (d) The number of foci in dissected lung metastases treated with PBS (control), PBA, PBA+HFMF, DON, and DON+HFMF intravenously at 14 days postinjection was quantified using ImageJ. Statistical significance was assessed using one-way ANOVA. Data represent mean ± SEM, *n* = 5. ****p < 0.001. (e) Biochemical indices of liver and kidney after 24 h of treatment. Statistical significance was assessed using one-way ANOVA. Data represent mean ± SEM, *n* = 3. (f) Survival patterns of animals with lung metastases following treatment with PBS (control), PBA+HFMF, DON, DON+HFMF, and DON+HFMF+αPD-1 (*n* = 6).

C57BL/6 mice were utilized to study the *in vivo* biodistribution of PBA and DON. Twelve days ago, metastases were induced by intravenous injection of GFP-B16F10 cells to prepare the lungs for particle injection. Then, QD (390 nm excitation; 600 nm emission)-labeled PBA and DON were administered to tumor-bearing mice via intravenous injection, as shown in Figure 6a. HFMF was applied on the second day following the DON treatment. Subsequently, major organs were collected and analyzed using the *In Vivo* Imaging System (IVIS) to evaluate the accumulation of nanocubes *in vivo* in Figure 6b. The findings revealed a higher accumulation of DON in the lung compared to PBA. However, the application of HFMF did not significantly enhance the tumor-targeting efficacy. This demonstrates the lung-targeting capabilities of negatively charged particles, and these particles were observed to be distributed throughout the lungs, often appearing as punctate fluorescent areas, indicating their internalization into phagocytes. It is worth noting that the fluorescence intensity of the DON group is almost twice that of the PBA group.

The mechanism of targeted lung metastasis is primarily attributed to exploiting the unique characteristics of the pulmonary vasculature and the specific conditions of the metastatic site for edge delivery.^68−70^ The pulmonary vasculature is characterized by a low-pressure, high-perfusion capillary network with a large surface area and a large number of small vessels. Particles with high density in the nanoparticle range are more likely to be marginalized because they experience greater lift forces that push them away from the core of high-velocity blood flow.^71,72^ These capillaries experience high shear rates, which promote the marginalization of circulating particles. Marginalization is the process by which particles in the blood flow move toward the vessel wall due to hydrodynamic forces. In the context of pulmonary microcirculation, nanoparticles, especially those in the size range of 100–200 nm, are driven to flow from the center to the periphery near endothelial cells.^73^

The enhanced permeability and retention (EPR) effect, a hallmark of tumor vasculature, further facilitates the targeting of lung metastases by nanoparticles.^74^ Tumors, including metastatic lung nodules, often have leaky vasculature and impaired lymphatic drainage, which allows nanoparticles that have migrated to the endothelial surface to extravasate or pass through the vessel walls more easily into the tumor tissue. This effect is particularly pronounced in the lungs, where the capillaries allow nanoparticles to more readily accumulate in areas of metastatic growth due to the EPR effect. On the other hand, the accumulation of particles in the liver may be due to the capture of nanoparticles by the mononuclear phagocytic system, which is abundant in organs such as the liver and spleen.

Figure 6c displays CLSM images of lung metastasis following the treatment of tumor-bearing mice with PBA, DON, and DON+HFMF at 24 h post injection. In the absence of treatment, lung images depicted green fluorescence indicative of GFP-B16F10 metastasis, while red fluorescence represented nanoparticles (NPs). Upon injection of QD-loaded nanoparticles via the tail vein, a significant number of particles exhibiting strong colocalization with lung metastasis were observed. Notably, a higher signal was detected in the DON+HFMF group compared to that in the other groups, suggesting that DON+HFMF treatment facilitated the penetration of lung metastasis and effectively surrounded the cell nuclei. Furthermore, other clearance organs, such as the liver and spleen, were evaluated to understand the distribution of particles. The results showed that particles accumulated predominantly in the liver across all groups, likely due to the activity of the reticuloendothelial system (RES), which efficiently clears foreign particles, including nanoparticles, from the bloodstream, as shown in Figure S8. Only a few particles were detected in the spleen.

### 2.8 *In Vivo* Mice Bearing Lung Metastasis Treated by DON and DON+HFMF

B16F10 cell metastasis represents an aggressive cancer variant that can rapidly spread to various organs. In this study, the efficacy of various treatments (PBA, DON, DON+HFMF, and DON+HFMF/aPD-1) was examined in mice with lung metastases (Figure S9). There were significantly fewer tumor nodules in the treatment group compared with the control group, which had approximately 800 tumor lesions. Specifically, the DON+HFMF and DON+HFMF/aPD-1 groups showed less than 160 and 20 nodules, respectively (Figure 6d). To assess metastasis to other organs, mice were intravenously injected with B16F10 cells and sacrificed 14 days later. Interestingly, no metastatic tumors were detected in any of the organs analyzed. Furthermore, respiratory monitoring of the mice after treatment showed no apparent complications. Histopathological examination after treatments with H&E staining revealed no serious thrombosis-related complications (Figure S10). It is important to note that this experiment followed the appropriate biosafety measures.

Liver and renal functions were evaluated after PBA, DON, DON+HFMF, and DON+HFMF/aPD-1 treatments (Figure 6e). ALT (alanine aminotransferase) and ALP (alkaline phosphatase) are key enzymes used to evaluate liver function. ALT, found mainly in the liver, helps in amino acid metabolism, and elevated levels in the blood typically indicate liver cell damage or disease. ALP, present in the liver, bones, kidneys, and bile ducts, is involved in processes like bile production and bone mineralization.^75^ The data show that therapeutic intervention has a minimal impact on these organs, demonstrating that antineoplastic drugs are safe and compatible with normal organ function. Furthermore, in combination with HFMF, cerium oxide plays a crucial role in tumor retention by acting as a catalase (CAT)-like agent. When encountering highly expressed H_2_O_2_ in the tumor microenvironment, cerium oxide oxidizes it into free radicals, which are critical in the induction of motility, thereby inhibiting metastatic growth.

Mice survival was followed for up to 60 days after treatment with PBS (control), PBA, DON, DON+HFMF, and DON+HFMF/aPD-1. The median survival of the control group was only 16 days, whereas the median survival of PBA- and PCA-treated mice was slightly prolonged (Figure 6f). Notably, mice treated with DON+HFMF and DON+HFMF/aPD-1 showed the most promising results, significantly extending the survival time. These findings suggest that DON+HFMF treatment induces antigen release through its chemotherapeutic effect on metastasis, thereby promoting immunotherapy. DON+HFMF integrates with the aPD-1 reservoir through catechol groups and, coupled with intensive HFMF-enhanced CDT, may trigger an increase in T lymphocytes at the metastatic site, ultimately improving survival outcomes.

### 2.9 Recruitment of T Cells to Pulmonary Metastases

**Figure 7:**
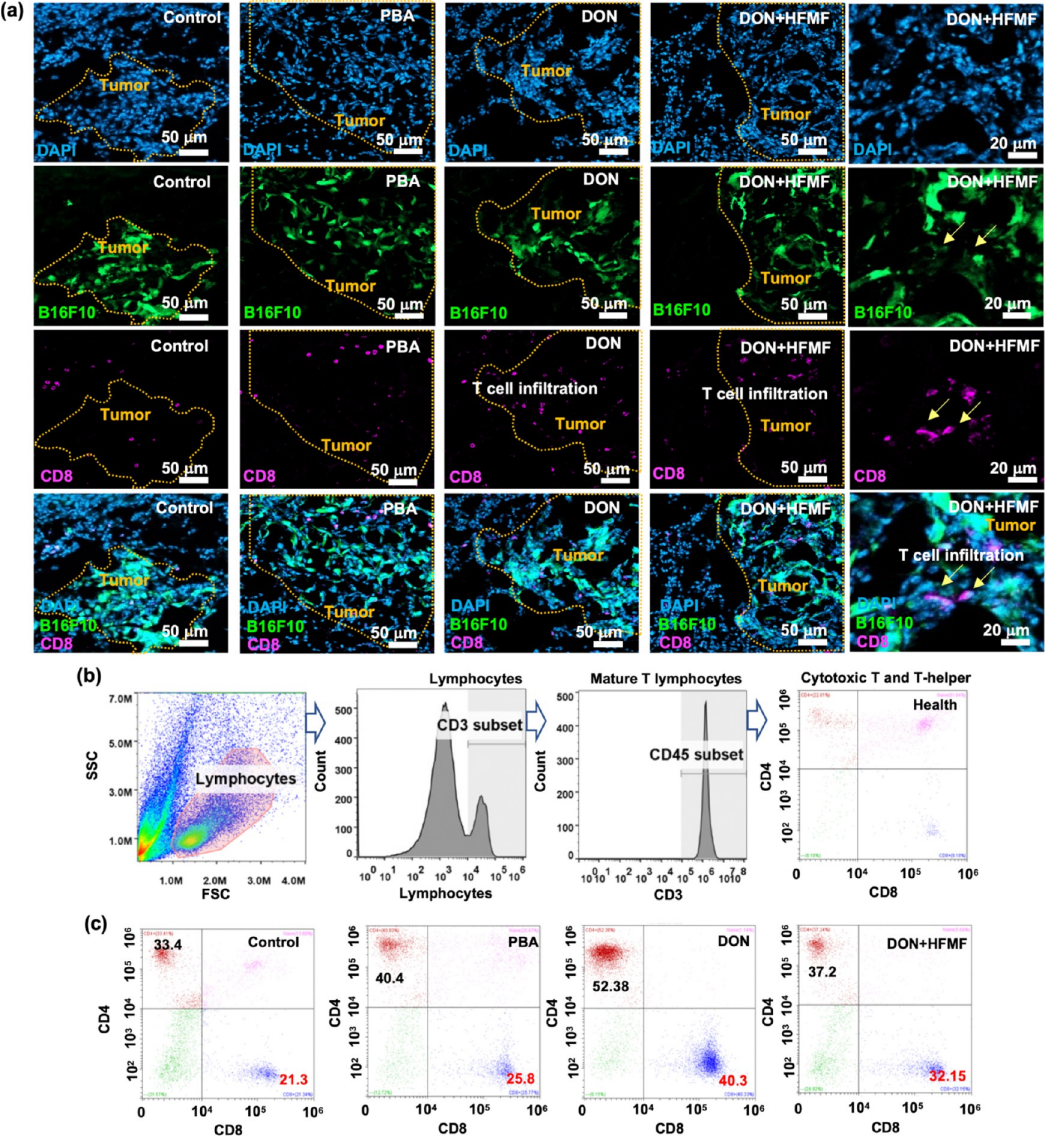
T cell infiltration in metastasis. (a) CLSM images of the lungs 24 h postintravenous injection of particles, along with measurements of CD4^+^ and CD8^+^ T cells. (b) Flow cytometry gating strategy for T cells. The lymphocyte population was selected based on SSC and FSC properties. Fluorescence gating for CD3, CD45, CD4, and CD8 was performed using fluorescence minus one (FMO) controls and single-staining compensation. The CD3^+^ population within the lymphocytes represents mature T lymphocytes, and further gating on CD45^+^ cells identify the cytotoxic T and T-helper cells. (c) Quantification of *in vivo* CD4^+^ and CD8^+^ T cells via flow cytometry analysis 24 h after control, PBA, DON, and DON+HFMF treatments.

Immune responses were studied by assessing the lymphocyte recruitment and infiltration at sites of lung metastasis. Mice with lung metastasis were intravenously injected with 100 μL of PBA, DON, and DON+HFMF, and the inhibition of lung metastasis was examined. After 24 h, lung tissue was extracted and stained with primary and secondary antibodies against CD4^+^ and CD8^+^ T cells. The research results shown in Figure 7a indicate that PBA, DON, and DON+HFMF effectively accumulate particles and chemotherapeutic drugs, promoting the infiltration and accumulation of T cells at the tumor site. Notably, DON+HFMF treatment showed the highest efficacy, with T cells showing enhanced penetration and accumulation within the tumor area, which was attributed to the synergistic effect of antigen capture and chemotherapy.

An *in vivo* flow cytometry gating strategy was employed to analyze CD4^+^ and CD8^+^ T cell populations in lung metastases treated with PBA, DON, and DON+HFMF. The findings revealed that the DON+HFMF group exhibited a higher expression of CD8^+^ single-positive T cells compared to the PBA group (Figure 7b,c). Statistical analysis was presented in Figure S11. Furthermore, the levels of immune factors such as tumor necrosis factor, interferon-γ (IFN-γ), and interleukin-10 (IL-10) in lung tissues treated with various samples were quantified using ELISA kits. The results presented in Figure S12 illustrated that the levels of IFN-γ and IL-10 in the DON and DON+HFMF groups were significantly elevated compared to the control group, suggesting the induction of an immune response.

### 2.10 Antigen Capture through DON and Immune Stimulation

**Figure 8:**
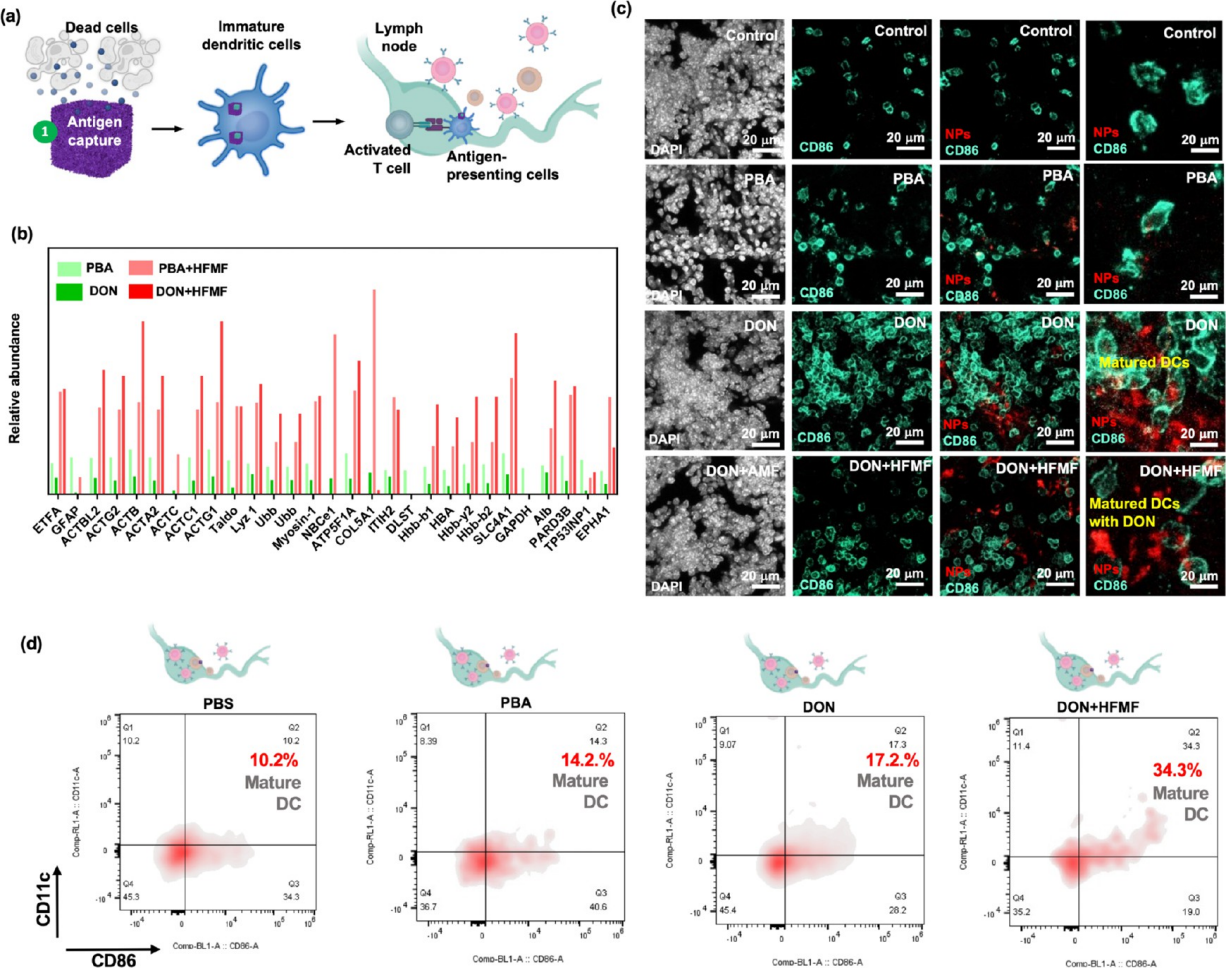
*In vivo* study of particle treatment in mice bearing B16F10 lung metastases. (a) The scheme of mechanism by which DON captures antigen and delivers it to antigen-presenting cells (APCs) such as dendritic cells (DCs). DCs then transport the antigen to lymph nodes, where they activate the immune system to induce a T-cell immune response. (b) Comparison of the percentage of antigen captured by PBA and DON, respectively. (c) CLSM image of dissected lymph node tissue 24 after injection. White, green, and red fluorescence represent nuclei stained with DAPI, DCs labeled with CD86, and nanoparticles labeled with QDs, respectively. (d) *In vivo* flow cytometry analysis of LN tissue dissected after 24 h post-treatment by PBA, DON, and DON+HFMF.

The effective capture of tumor-associated antigens (TAAs) by PBA and DON is primarily facilitated by the porous structure and hydrophobic characteristics of the particles. Calcination is a process involving the heating of materials to high temperatures under controlled atmospheres. These transformations augment their surface properties by enhancing the surface area and modifying the surface chemistry. Organic or volatile compounds within the material are eliminated during calcination, resulting in a more porous and reactive surface conducive to molecular adsorption.^76,77^ This heightened surface area provides increased binding sites for molecules, thereby enhancing the adsorption capacity. Furthermore, calcination-induced crystalline rearrangements or phase transformations further optimize surface properties, favoring interactions with molecules for adsorption. Consequently, the synthesized particles can capture antigens, facilitating recognition by DC and subsequent delivery to lymph nodes (LN) (Figure 8a).

To assess the release of neoantigens and damage-associated molecular patterns (DAMPs) from B16F10 cells by particle treatment, detailed experimental procedures are outlined in the protocol (Figure S13). The released antigen was captured by PBA and DON and analyzed using liquid chromatography-mass spectrometry (LC-MS/MS, Orbitrap Elite^TM^ hybrid ion trap-Orbitrap mass spectrometer, Thermo Fisher, USA). Notably, a comprehensive spectrum of well-known proteins, including more than 50 highly distinguishable proteins, was observed on PBA and DON (Figure S14). DAMPs are endogenous molecules released under cellular stress and are potent activators of immune responses (Figure S14). Several distinctive features were found among the released antigens. For example, the membrane-bound protein Ephrin, known for its role in cell adhesion and migration, has been implicated in cancer, with EphA1 being particularly associated with lung and lymph node metastasis.^78^ Additionally, actin is critical for cellular structural support, and when released from dying cells, it is recognized by the DNGR-1 receptor as a DAMP.^79^ In addition, ubiquitin is a heat-stable protein with important regulatory functions in eukaryotic cells, mainly promoting the degradation of intracellular proteins. These released antigens are thought to be endogenous antagonists of DAMPs that modulate immune responses.

DCs are the most potent antigen-presenting cells (APCs), adept at efficiently presenting antigens and enhancing immune responses against tumors. Immature DCs have a strong migration ability and can be recruited to tumor sites through the presentation of DAMPs such as EphA1 and mature ubiquitin antigens. Therefore, DC recruitment by PBA, DON, and DON+HFMF antigen capture was studied *in vivo*. The effect of particles on the *in vivo* recruitment of DCs to lung metastases was evaluated in mice bearing B16F10 lung metastases. To facilitate tracking, PBA, DON, and DON+HFMF are premarked with DiI. 24 h after injection, lymph nodes (LNs) and spleens were dissected from the animals, and DCs and T cells in the LNs were quantified. Figure 8c depicts a CLSM image of LN tissue stained with CD86, a marker indicative of DC upregulation and immune activity. White, green, and red fluorescence correspond to the nuclear staining of DAPI, DC, and nanoparticles (NPs), respectively. The results showed the presence of four distinct groups in LNs, all of which showed strong expression of CD86. The accumulation of DCs within LNs can be attributed to the antigen-capturing particles, of which DON+HFMF showed significant adhesive properties and demonstrated efficacy in adsorbing and delivering antigens. This facilitated the identification of DCs and thus elucidated the mechanism by which these particles promote DC accumulation. Additionally, we employed *in vivo* flow cytometry to assess the maturation of DCs in the LNs after administering PBA and DON (Figure 8d**)**. The quantification results were given in Figure S15. The DON+HFMF group displayed a significantly higher expression of CD86^+^CD11c^+^ compared to the PBA groups, suggesting the efficient maturation of DC cells by DON+HFMF.

To further mimic this reprogramming of DCs, tumor cells were lysed, and the resulting culture medium was cocultured with DON. To evaluate the antigens captured by DON, the particles were analyzed using liquid chromatography–mass spectrometry (Figure S16a). The results closely matched those from cell-based studies, revealing several distinctive features among the captured antigens, including membrane-bound proteins such as Ephrin and heat-stable proteins with regulatory functions in eukaryotic cells that promote the degradation of intracellular proteins. These released antigens are believed to act as endogenous antagonists of damage-associated molecular patterns (DAMPs), thereby modulating immune responses.

To further investigate the ability of antigen-loaded DON to activate DC maturation, the particles were administered via subcutaneous injection. After 24 h, the particles accumulated in the lymph nodes, which were then collected and analyzed. As shown in Figure S16b, CLSM images of the lymph nodes demonstrated DC uptake and maturation. These findings indicate that an antigen-loaded DON can effectively activate DCs. This conclusion is further supported by consistent results obtained via flow cytometry. The results confirm that the antigen-loaded DON particles can engage with DCs *in vivo*. This engagement is a crucial step in the immune activation cascade, leading to the presentation of tumor antigens and the potential activation of cytotoxic T cells, which target tumor cells. Additionally, the maturation of DCs involves the upregulation of costimulatory molecules and cytokine production, further enhancing the immune response. These results provide evidence that antigen-loaded DON can serve as a potent immunotherapeutic agent by facilitating the capture and presentation of tumor antigens and promoting DC maturation, thereby enhancing antitumor immunity.

**Figure 9:**
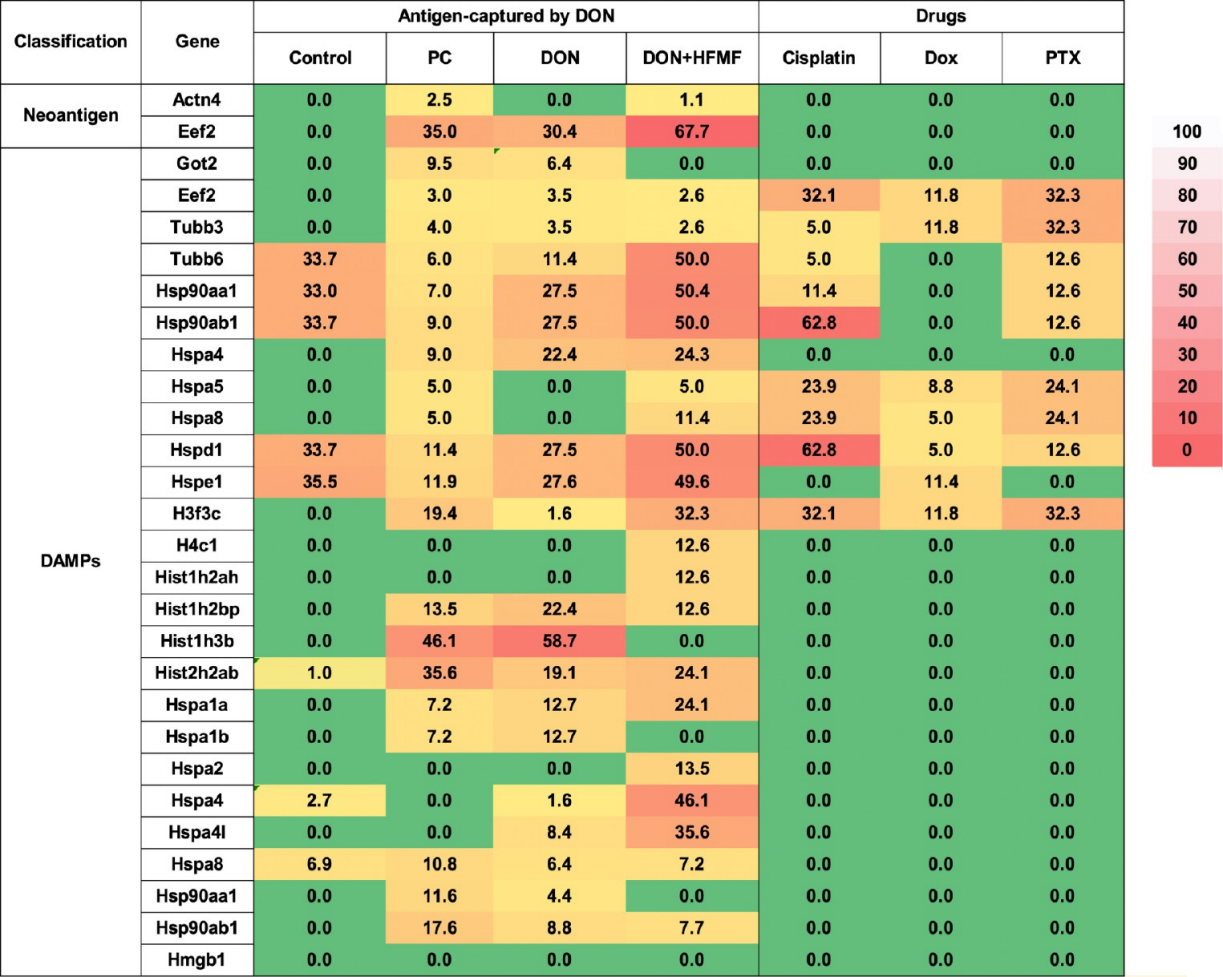
Antigen release after PC, DON, DON+HFMF, and chemodrug-treated cells.

The heat map showcased in Figure 9 unveils the antigen release dynamics of B16F10 cells, shedding light on the expression patterns of secreted chemokines following treatment with the chemodrugs PBA and DON. Notably, compared to cells treated solely with chemodrugs, those treated with PBA and DON displayed heightened expression of DAMPs overall. DAMPs are internal molecules released amidst cellular stress. Eef2, a membrane-bound protein renowned for its roles in cell adhesion and migration, has been implicated in cancer, particularly linked to lung and lymph node metastasis. Moreover, Tubb3 or Tubb6 assumes a pivotal role in providing structural support to cells; upon release from dying cells, it activates receptors as DAMPs. Additionally, Hsp and Hspa, recognized as stable proteins, undertake crucial regulatory functions within eukaryotic cells, predominantly aiding in the degradation of intracellular proteins. These released antigens are postulated to function as endogenous antagonists to DAMPs, thus modulating immune responses.

## 3 Discussion

In cancer immunotherapy, a critical component involves the efficient capture and delivery of tumor-associated antigens (TAAs) to lymph nodes, where they activate the immune system. This activation primarily occurs through the stimulation of APCs, which initiate the immune response by presenting TAAs to T cells. In this study, we developed DON, composed of CeO_2_ and IO, designed to overcome these challenges in immunotherapy and DC programming. Following systemic administration, DON accumulates in the lungs via the margination effect and penetrates lung metastases through uptake by alveolar endothelial cells and leukocytes. Once at the tumor site, exposure to HFMF in combination with CDT triggers a catalytic cycle that enhances the decomposition of H_2_O_2_, leading to the generation of ROS. This oxidative stress amplifies the redox cycling of Fe^3+^/Fe^2+^ and Ce^3+^/Ce^4+^, enhancing its recyclability and triggering cancer cell apoptosis.

The results of this study highlight the synergistic effects of cerium oxide and HFMF in enhancing ROS production. This enhanced ROS generation demonstrates significant potential for efficient tumor elimination. According to Kelvin’s force laws, the application of a higher magnetic field results in a greater Kelvin force, which in turn accelerates mass transfer. This principle is evident in the group treated with HFMF, which exhibited elevated levels of oxOPD, indicating increased ROS production. The higher oxOPD levels can be attributed to the enhanced interfacial electron charge transfer between the DON and H_2_O_2_ under the influence of HFMF. This interaction accelerates the decomposition of H_2_O_2_ and promotes the generation of hydroxyl radicals, a crucial factor in CDT and the oxidative-stress-mediated killing of cancer cells. The effective ROS generation through redox cycles, combined with the magnetic field’s influence, further supports the potential of this approach in improving cancer treatment outcomes.

Another feature of this system is its ability to promote lysosomal escape and inhibit autophagy, processes critical for the induction of immunogenic cell death. The resulting release of TAAs is captured by the porous structure of DON, which acts as an antigen reservoir and facilitates prolonged antigen presentation. This not only sustains immune activation but also suppresses tumor metastasis by ensuring the continuous release of TAAs at the tumor site. Additionally, the captured antigens recruit more dendritic cells, enhancing the immune response mediated by both CD4^+^ and CD8^+^ T cells.

This study assessed the release of neoantigens and DAMPs from B16F10 cells following treatment with the particles. Using LC-MS/MS, a broad range of well-known proteins was captured by PBA and DON. Among the identified proteins were notable DAMPs, such as membrane-bound Ephrin, actin, and ubiquitin. These findings suggest that the released antigens and DAMPs could be leveraged to enhance immune activation and antitumor responses, making them valuable targets for future immunotherapeutic strategies.

The dual functionality of DON—catalytically driving cancer cell apoptosis and simultaneously capturing and delivering antigens—presents a promising strategy for enhancing the efficacy of cancer immunotherapy. By improving antigen retention, stimulating immune responses, and suppressing metastasis, this system holds significant potential for future therapeutic applications, particularly in combating difficult-to-treat metastatic cancers.

## 4 Conclusion

In summary, this study introduces dual catalytic oxide nanozymes (DON) capable of serving as both a dual catalyst and an inducer of T cell infiltration, thereby facilitating immune therapy and mediating dendritic cells. Intravenous administration of DON enhanced tumor accumulation through targeted margination. Upon reaching the tumor site, DON incorporates CeO2-coated iron oxide nanocubes, functioning as a programmed peroxide mimetic within cancer cells, thus promoting sustained ROS generation and depleting intracellular glutathione, known as chemodynamic therapy (CDT). Under HFMF irradiation, CDT accelerates H_2_O_2_ decomposition, leading to increased production of reactive oxygen species, while also promoting sustainable cycling between Fe^3+^/Fe^2+^ and Ce^3+^/Ce^4+^ species on an active surface rich in Fe(II) ions. Concurrent hyperthermia further augments this process, facilitating tumor release of tumor-associated antigens, including neoantigens and damage-associated molecular patterns. Subsequently, the porous structure of DON acts as an antigen transporter, delivering autologous tumor-associated antigens to DCs and sustaining immune stimulation. The combination of catalytic DON with an immune checkpoint inhibitor (anti-PD1) in lung metastases effectively suppresses tumors and significantly prolongs survival.

## 5 Experimental Section

### 5.1 Materials

Polyvinylpyrrolidone (PVP, Sigma-Aldrich, CAS Number: 9003-39-8), potassium ferricyanide (J.T. Baker, CAS Number: 13746-66-2), hexamethylenetetramine ((CH2)6N4, MeAlfa Aesar, CAS Number: 100-97-0), Cerium(III) nitrate hexahydrate (Acros Organics, CAS Number: 10294–41–4), hydrogen peroxide (Honeywell, CAS Number: 7722-84-1), o-phenylenediamine (Sigma, CAS Number: 95-54-5), resiquimod (Taiclone, CAS Number: 144875-48-9).

### 5.2 Synthesis of PB, PC, PBA, and DON

Following established methodologies, Prussian blue nanocubes (PB) were synthesized as outlined: 3 g of polyvinylpyrrolidone (PVP) and 226.7 mg of potassium ferricyanide were dissolved in 40 mL of deionized water. Subsequently, 35 μL of concentrated hydrochloric acid was introduced into the solution, and the mixture was allowed to react for 20 h at 80 °C to yield PB. The resulting PB underwent extensive washing with deionized water and ethanol, followed by drying in a 60 °C oven.

For the cerium oxide coating, 50 mg of PB was dispersed in 40 mL of a 50% ethanol solution. To this solution, 75 mg of Ce(NO_3_)_3_·6H_2_O and 200 mg of (CH_2_)_6_N_4_ were added, and the mixture was agitated at 70 °C for 2 h. The cerium oxide-coated PB (PC) was then subjected to centrifugation at 12,000 rpm to remove the supernatant, followed by three washes with deionized water. The resulting product was designated as PC. To obtain magnetic porous particles, PB or PC underwent annealing at 450 °C under a nitrogen environment for 6 h, leading to the conversion into Annealed PB (PBA) or annealed cerium oxide-coated PB (DON), respectively.

### 5.3 Material Characterization

To analyze the morphologies and elemental mapping of nanoparticles, we utilized a high-resolution thermal field emission scanning electron microscope (HRFEG-SEM, JSM-7610F, JEOL, Japan) and field emission transmission electron microscopy (JEM-F200, JEOL Ltd.). For SEM analysis, all samples were dried on silicon wafers and coated with a thin film of gold on the surface to increase their conductivity during intensive electronic sputtering. For the preparation of TEM samples, the nanoparticles were dispersed in ddH2O and dropped onto a copper grid with 200 meshes. After drying the grid in the oven overnight, we observed the morphologies of the nanoparticles under TEM to obtain representative images. By using the EDS elemental mapping system of TEM, we can also obtain the element mapping results of the nanoparticles.

The size and zeta potential of nanoparticles were analyzed by dynamic light scattering (DLS, TreksizerNano 90 Zeta, TRK). Samples were diluted with ddH2O in a glass cuvette, and the distribution of nanoparticles was measured through a laser that detected the Brownian motion of particles and transformed the signal into particle size. As for the surface and pore properties of nanoparticles, a surface area and pore size distribution analyzer (BET, ASP 2020, Micromeritics) was used to determine the surface area and pore size of the nanoparticles. The magnetism of the nanoparticles was determined by the superconducting quantum interference device magnetometer (SQUID, MPMS-3, USA Quantum Design). Utilizing thermal analyzers (TGA, 2-HT, Mettler-Toledo), we analyzed the nanoparticles with increasing temperature to determine the physicochemical characteristics of the nanoparticles. Furthermore, an X-ray Powder Diffractometer (XRD, APEX DUO, Bruker) was used to determine the crystallites and structure of the nanoparticles, and a High-Resolution X-ray Photoelectron Spectrometer (HRXPS, PHI Quantera II, ULVAC-PHI) was used to detect the elements and the bonds inside the nanoparticles. Finally, we used UV–vis spectrometry (SP-8001, Metertech) to detect the catalytic activity of nanoparticles and the ability of bovine serum albumin (BSA) capture by the change of the adsorption peak.

### 5.4 Catalytic Effects

The catalytic efficiency of DON at a concentration of 20 g L^–1^ was investigated for the degradation of rhodamine B. In these experiments, DON was introduced into a dye solution with a concentration of 15 mg L^–1^. Hydrogen peroxide (H_2_O_2_) was then added using a pipette to initiate the degradation process. The experiments were conducted at room temperature under an external high-frequency magnetic field (HFMF). The HFMF had a fixed sinusoidal frequency of 50 kHz, and its magnetic strength was adjusted by varying the input of the alternating current. The maximum magnetic strength (Bmax) was directly measured by using a magnetometer. Samples of approximately 1 mL were taken at specific time intervals and analyzed by using fluorescence spectroscopy. The apparent kinetic rate constant (*k*_obs_) was calculated by using ln(*C*_0_/*C*_t_) = *k*_obs_, where *C*_0_ is the original concentration of the dye and *C*_t_ is the concentration of the dye at time *t*.

For the catalytic activity of nanoparticles, the chemical compound o-phenylenediamine (OPD) as a ROS detector was used. First, the nanoparticle solution (PBA and DNA) is dispersed in PBS solution (pH 5.5), and we will use OPD as a substrate in PBS solution (pH 5.5) containing 100 μM H_2_O_2_. The OPD solution is then added to the nanoparticle solution to mix and generate ROS. OPD is oxidized by ·OH after contact with ROS, and the color of the solution obviously changes from transparent to yellow. Finally, we measured the characteristic absorption peaks of oxidized OPD through a UV–visible spectrophotometer.

For *in vitro* studies of ROS production, we used commercial detection kits to verify the production of specific ROS (·OH). As per the guidelines, a 250x OH580 stain stock should be prepared by first mixing 50 μL of DMSO into the included OH580 vial. In terms of experiments, B16F10 cells were placed in a confocal culture dish at a concentration of 2 × 10^5^ cells/mL and cultured in a 37 °C, 5% CO_2_ incubator for 24 h. Then, the OH580 staining working solution was prepared by adding 25 μL of 250x OH580 stain stock solution to a centrifuge tube containing 10 mL of assay buffer. After removing the culture medium from the confocal dish, we replaced it with 1 mL of OH580 staining working solution and soaked the cells in this solution for one h in the incubator. Then, 1 mL of warm PBS buffer dissolved in 100 μM H_2_O_2_ and the same dose of different nanoparticles were added to each confocal dish as a treatment and incubated for another 1 h. Wash three times with PBS and stain the nuclei with prepared Hoechst 33342 staining buffer. Finally, 1 mL of assay buffer was used to replace the nanoparticle solution to preserve the sample, and the sample was observed through CLSM with 540/590 excitation/emission to detect the state of ·OH.

In addition, a glutathione (GSH) consumption test of different nanoparticles was designed to verify the catalytic function of the nanoparticles. First, nanoparticles at a concentration of 200 μg/mL were mixed with GSH (20 mM) in a PBS solution. We added 0.4 mM 5,5′-dithio-bis(2-nitrobenzoic acid) (DTNB) to the above solution to detect the −SH group of GSH. We observed a change in absorbance around 412 nm and recorded the absorbance using a UV–visible spectrophotometer.

### 5.5 *In Vitro* Studies of PB, PC, PBA, and DON

The B16F10 cells and GFP-carrying B16F10 (GFP-P2A-NanoLuc B16F10) were cultured in Dulbecco’s Modification of Eagle’s Medium (DMEM) supplemented with 10% fetal bovine serum (FBS) and 1% penicillin-streptomycin (Gibco, 15140122) at 37 °C in a 5% CO_2_ incubator. Cytotoxicity assessment of nanocubes employed a resazurin-based assay (Thermo, PrestoBlue A13261). Briefly, 100 μL of cells at a concentration of 10^5^ cells/mL were seeded into a 96-well plate and cultured overnight. On the following day, varying concentrations of nanocubes were added, and the samples were incubated for an additional day. Subsequently, 20 μL of PrestoBlue was added, and the signal was developed as per the supplier’s instructions. Fluorescence signals were detected by using a microplate reader.

For assessing nanocube distribution and cellular uptake, Quantum Dots (QDs, absorption maximum 390 nm; emission maximum 600 ± 10 nm) were employed to label the nanocubes. QDs were dissolved in chloroform, mixed with nanocubes, and subjected to overnight incubation. Following the removal of excess dye via centrifugation at 12,000 rpm for 10 min and triple washing with purified water, nanocubes were prepared for subsequent procedures. Cells were plated into 6-well or 24-well plates with coverslips for flow cytometry or confocal imaging. After 16 to 18 h of incubation, 200 μg/mL of specified labeled nanocube groups were introduced and further incubated for 24 h. Coverslip cells were fixed with 3.7% paraformaldehyde and stained with phalloidin and DAPI. In the case of the flow cytometry assay, cells were detached from the plate using trypsin-EDTA and resuspended in PBS.

### 5.6 DC2.4 Cell Culture

DC2.4 cells, a murine dendritic cell line, are cultured under standard conditions to maintain their viability and functionality. Begin by quickly thawing a frozen vial of DC2.4 cells in a 37 °C water bath. Transfer the cells to a sterile 15 mL conical tube containing 10 mL of prewarmed complete culture medium, which typically consists of RPMI-1640 supplemented with 10% fetal bovine serum (FBS), 1% penicillin-streptomycin, and 50 μM β-mercaptoethanol. Centrifuge the cells at 300*g* for 5 min, discard the supernatant, and resuspend the cell pellet in fresh complete medium. Plate the cells in a *T*-75 flask at a density of 0.5–1 × 10^6^ cells/mL and incubate at 37 °C in a humidified atmosphere with 5% CO_2_.

For routine maintenance, the medium is changed every 2–3 days, and the cells are monitored for confluency. When the cells reach 70–80% confluency, they are subcultured by gently detaching with a cell scraper or pipetting and then reseeded at a lower density. For experimental setups, seed DC2.4 cells in appropriate culture vessels (e.g., 6-well plates) and allow them to adhere and recover for 24 h before any treatments.

### 5.7 Antigen Capture by PCA and DON

To evaluate the ability to capture antigens, the sodium dodecyl sulfate polyacrylamide gel electrophoresis (SDS-PAGE) method by MS Spectrometry (LC/MS/MS, Orbitrap Elite MS006200, Thermo Fisher Scientific) was applied. First, we needed to prepare the stacking gel and separating gel, and the materials that we used included H2O, 30% acrylamide mix, 1.5 M Tris buffer (pH 8.8), 1 M Tris buffer (pH 6.8), 10% SDS, 10% ammonium persulfate, and TEMED. According to different recipes, we could make different percentages of separating gel, and we chose 10% gel as the separating gel.^43^

After preparing the gel, the B16F10 cells were cultured in a 6-well plate at a concentration of 10^5^ cells per well for 24 h to allow the cells to adhere to the well. Once the cells adhered to each well, they were washed with PBS three times, and DMEM without 10% fetal bovine serum (FBS) and 1% penicillin–streptomycin was added to each well to ensure that the samples would not interfere with other proteins. Then, the 6-well plate was incubated in a 37 °C incubator with 5% CO_2_ for 48 h. After incubating for 48 h, we replaced the medium with DMEM containing the appropriate concentration of chemodrug or nanoparticles, which could cause more than 50% apoptosis, and returned the well plate to the incubator for 24 h. Afterward, we removed the supernatant and added it to Amicon Ultra Centrifugal Filters, centrifuging twice at 5,000*g* for 5 min at 4 °C. Finally, the centrifuged liquid could be used for the SDS-PAGE experiment. In addition, we added and mixed nanoparticles into the centrifuged liquid to validate that our nanoparticles could capture antigens.

Next, we prepared the samples for LC/MS/MS. Sample buffer was added in an appropriate volume (sample: sample buffer = 10:2) to the different groups of supernatants and incubated at 95 °C for 10 minutes using a dry bath incubator. Then, the 10 μL samples were ready to be loaded into a gel, and the electrophoresis cell was connected to the power supply. Subsequently, we set the fixed current at 30 mA and waited for the samples to reach one centimeter from the separating gel. After removing the gel from the plate, we utilized Coomassie Blue to stain the gel using a microwave for 30 s. The gel immersed in Coomassie Blue was shaken at 50 rpm for 15 min using an orbital shaker. The destain solution was used in the microwave for 30 s after finishing the shaking process. Finally, we cut the gel into an area of one square centimeter and kept them at 4 °C.

### 5.8 *In Vivo* Biodistribution Study

The animal experiments involved the use of C57BL/6 mice, purchased from BioLASCO Co., Ltd., aged between 8 and 10 weeks. The lung metastasis model was established by intravenously injecting 5 × 10^5^ of GFP-P2A-NanoLuc B16F10 cells via the tail veins. Twelve days postinoculation, doxorubicin-labeled nanocubes were intravenously administered to the mice. The following day, the mice were euthanized, and major organs were isolated. Biodistribution images were captured using the IVIS Spectrum *In Vivo* Imaging System (PerkinElmer). The lungs were subsequently fixed in 4% paraformaldehyde overnight at 4 °C.

The fixed tissues underwent frozen sectioning after embedding in the OCT compound (Sakura, 4583). Subsequent immunofluorescence staining involved the use of a 500-fold diluted anti-CD31 antibody (BD Pharmingen, 550274) and a 500-fold diluted secondary antibody (Abcam, ab150155) to label normal endothelial cells. Confocal laser scanning microscopy (CLSM) images were acquired to evaluate the distribution of the nanocubes within the lung.

### 5.9 Dendritic Cells Induced Immune Response in Lymph Nodes

The immune response triggered by nanocubes in B16F10 tumor-bearing mice was assessed. Nanocube treatments were administered on days 7, 10, and 13 post-tumor inoculation. After 15 days, the mice were euthanized, and the cervical lymph nodes were isolated for subsequent immunofluorescence staining. DCs were labeled using a 500-fold diluted anti-CD86 antibody (Abcam, ab119857) and a 1000-fold diluted secondary antibody (Abcam, ab150160). CLSM images were acquired to evaluate the colocalization of nanocubes and DCs.

## Supporting Information Available

Adsorption of proteins into different polymer matrices (Figure S1). XPS spectrum (Figure S2) and Brunauer–Emmett–Teller (BET) analysis (Figure S3). CLSM images of DCs incubated with PBA and DON (Figure S4). Cell viability of HUVEC and L929 treated with DON at various concentrations with and without subjecting to HFMF (Figure S5). CLSM images of *in vitro* assessments of anti-CRT (Figure S6). *In vitro* flow cytometry analysis of DCs after post-treatment by antigens@DON (Figure S7). CLSM images of liver and spleen of mice (Figure S8). Nanoparticle and anti-PD1 treatment regimens for B16F10 cell metastasis (Figure S9). H&E (hematoxylin and eosin) staining of main organs of mice (Figure S10). The quantification of CD8^+^ single positive T cells in T lymphocytes (Figure S11). The immune factor concentrations of IFN-γ and IL-10 in lung tissues (Figure S12). The experimental procedures of analysis of the neoantigens and damage-associated molecular patterns (Figure S13). The release of neoantigens and damage-associated molecular patterns (DAMPs) from B16F10 cells by particles treatment (Figure S14). The statistical result for DCs maturation (Figure S15). Tumor-released antigens captured by DON and IVIS and CLSM image of dissected lymph node tissue 24 h after injection (Figure S16) (PDF)
