# Supplementary material for: Reprogramming Dysfunctional Dendritic Cells by a Versatile Catalytic Dual Oxide Antigen-Captured Nanosponge for Remotely Enhancing Lung Metastasis Immunotherapy
Source: ACS Nano. 2024 Dec 31;19(2):2117–35. doi: 10.1021/acsnano.4c09525 (PMC11760334; doi:10.1021/acsnano.4c09525)
Supplement: Supplementary file 1 — nn4c09525_si_001.pdf [file nn4c09525_si_001.pdf]

Supporting Information

Reprogramming Dysfunctional Dendritic Cells by a Versatile Catalytic Dual Oxide Antigen Captured Nanosponge for Remotely Enhancing Lung Metastasis Immunotherapy

Min-Ren Chiang[1], *Chin-Wei Hsu* [1], *Wan-Chi Pan* [1], *Ngoc-Tri Tran* [1], *Yu-Sheng Lee* [1], *Wen-Hsuan Chiang* [2], *Yu-Chen Liu* [3], *Ya-Wen Chen* [4], *Shih-Hwa Chiou* [5], *Shang-Hsiu Hu*[1,*]

[1] Department of Biomedical Engineering and Environmental Sciences, National Tsing Hua University, Hsinchu 300044, Taiwan

[2] Department of Chemical Engineering, National Chung Hsing University, Taichung 402, Taiwan

[3] Laboratory for Human Immunology (Single Cell Genomics), WPI Immunology Frontier Research Center, Center for Infectious Disease Education and Research (CiDER), Osaka University, Osaka, 565-0871, Japan

[4] National Institute of Cancer Research, National Health Research Institutes, Miaoli County, 35053, Taiwan

[5] Institute of Pharmacology, College of Medicine, National Yang Ming Chiao Tung University, Taipei, 112304, Taiwan

[6] Department of Medical Research, Taipei Veterans General Hospital, Taipei, 112304, Taiwan

E-mail addresses: shhu@mx.nthu.edu.tw

Experimental Section

In vitro study of HUVEC and L929

To culture HUVEC, begin by preparing tissue culture flasks coated with a gelatin to promote cell adhesion. HUVECs are then seeded in flasks containing specialized endothelial cell growth medium, such as EGM-2, which contains essential growth factors like VEGF, FGF, and heparin. The cells are typically incubated at 37°C in a humidified atmosphere with 5% CO_2.

Medium should be replaced every 2–3 days, and cells are passaged when they reach 70-80% confluency. For passaging, the cells are washed with PBS, detached using a mild trypsin-EDTA solution, and reseeded into new coated flasks at an appropriate density. Care must be taken to maintain sterility and prevent contamination, as endothelial cells are sensitive to culture conditions.

L929 cells are grown in standard Dulbecco's Modified Eagle Medium (DMEM) supplemented with 10% fetal bovine serum (FBS) and antibiotics like penicillin-streptomycin. The cells are incubated at 37°C in a 5% CO2 environment, with medium changes every 2–3 days. L929 cells are more robust and can be passaged when they reach around 80% confluency. Similar to HUVECs, they are detached using trypsin-EDTA, centrifuged, and then reseeded at the desired density for further experiments.

In vivo analysis of T lymphocytes associated immune responses

GFP-P2A-NanoLuc B16F10 tumor-bearing mice were established by inoculating GFP-P2A-NanoLuc B16F10 cells, following a previously described process. Nanocube treatments were administered on days 7, 10, and 13 post-tumor inoculation. After 15 days, the mice were euthanized, and lung tissues were isolated for frozen sectioning. Staining for $CD8^+$ cells was performed using a 500-fold diluted anti-CD8 antibody (Abcam, ab217344) and a 500-fold diluted secondary antibody (Abcam, ab150075). CLSM images were taken to evaluate the T lymphocytes-associated immune responses.

Flow cytometry for T cell immune responses

Flow cytometry was employed to quantify the cytotoxic T cells and helper T cells following the administration of nanocubes and anti-PD1 treatment. Subsequent to the treatment procedure as

previously described, the lungs were isolated and enzymatically digested using a mixture of 0.1 mg/mL collagenase (Sigma, C0130), 1 μg/mL DNase (Sigma, DN25), 6.6 μg/mL dispase I (Sigma, D4818), and 3 mL RBC lysis buffer (Thermo, 00-4333-57) in HBSS buffer (Sigma, H8264) for 60 minutes at room temperature with continuous shaking at 190 rpm. The resulting tissue solutions were then filtered through 70 μm cell strainers to eliminate undissociated tissues. To mitigate non-specific antibody binding and block Fc receptors, 50 μL goat serum and 10 μL Fc Blocker (BD Pharmingen, 553142) were added to the samples and incubated at room temperature for 45 minutes.

Subsequently, the cell solution was distributed into several flow tubes for antibody staining. PE/Cyanine7 anti-mouse CD45 antibody (Biolegend, 103114, dilution ratio: 1:500), FITC anti-mouse CD3ε antibody (Biolegend, 100306, dilution ratio: 1:500), PE anti-mouse CD4 antibody (Biolegend, 100408, dilution ratio: 1:500), and APC anti-mouse CD8a antibody (Biolegend, 100712, dilution ratio: 1:500) were employed to identify cytotoxic T cells and helper T cells. Following a 60-minute incubation on ice with the antibodies, the stained cells were centrifuged at 1550 rpm for 5 minutes to remove free antibodies and then resuspended in 1 mL of PBS. Finally, the samples were analyzed using the Attune Nxt flow cytometer to determine the ratio of cytotoxic T cells to helper T cells.

In vivo study of antitumor

The B16F10 tumor-bearing mice were established following the previously described procedure. Nanocubes were intravenously injected on days 7, 10, and 13 post-tumor inoculation. In the groups subjected to combined therapy with immunotherapy, anti-PD1 antibodies were administered to mice on days 8, 11, and 14 post-tumor inoculation. On day 15, mice were sacrificed, and tumor nodules were counted under a dissecting microscope.

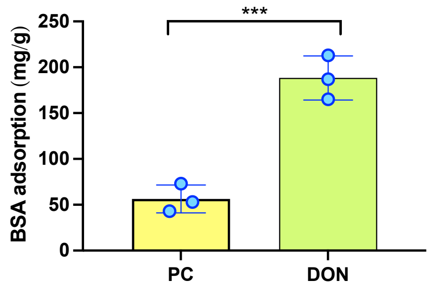

Figure S1. Adsorption of proteins into different polymer matrices. Bovine serum albumin (BSA) was used as a model protein for adsorption testing. (n = 3; mean ± s.d.; *** p < 0.005; one-way ANOVA with Tukey's multiple comparison test).

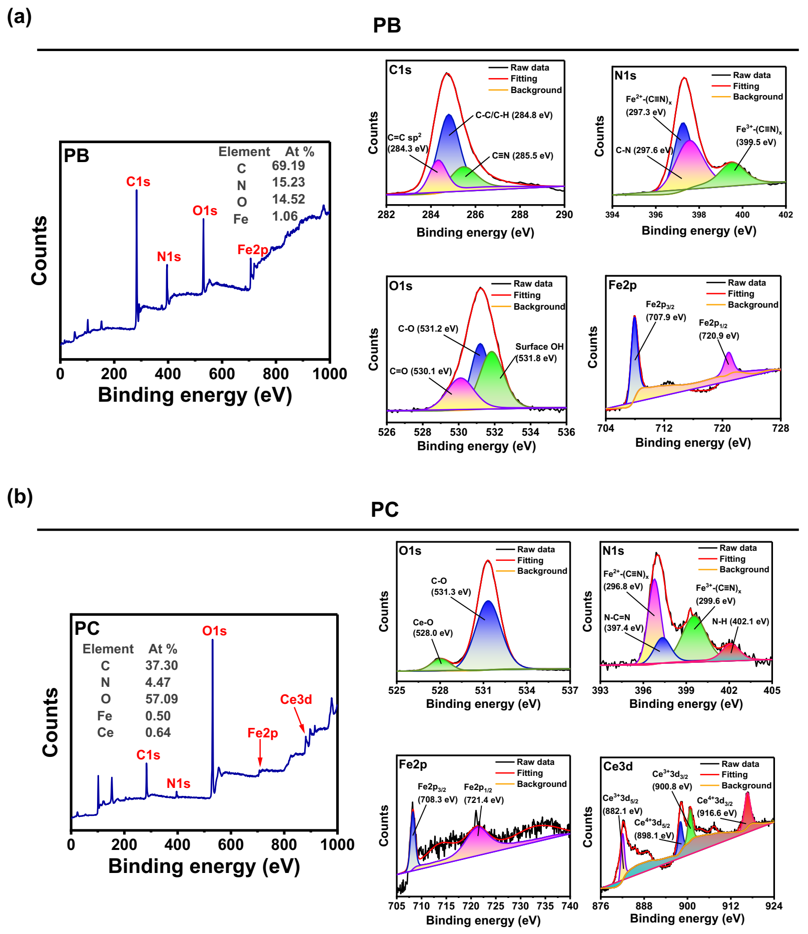

Figure S2. XPS spectrum of C1s, O1s and Fe2p of (a) PB and (b) PC.

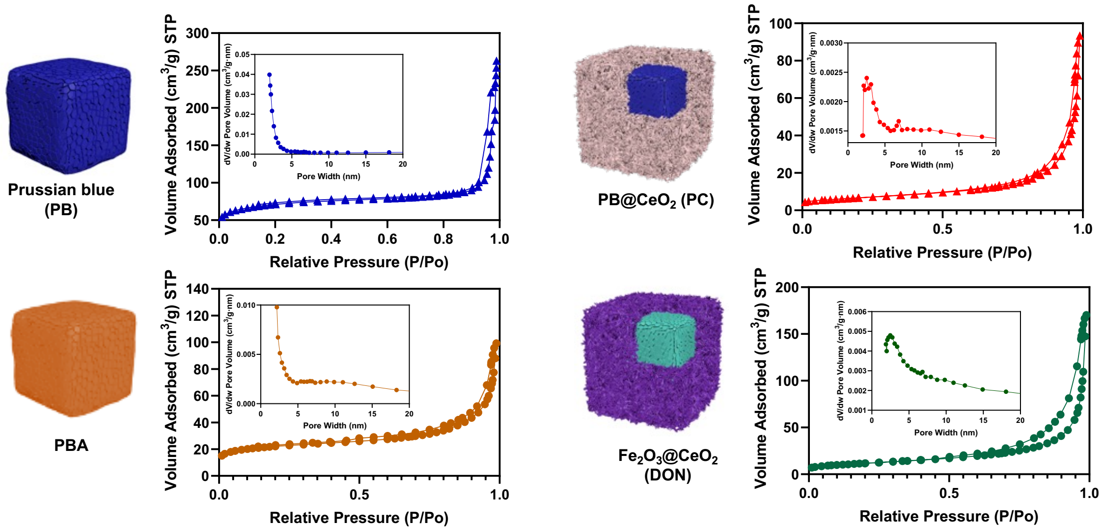

Figure S3. Brunauer-Emmett-Teller (BET) analysis of PB, PC, PBA, and DON.

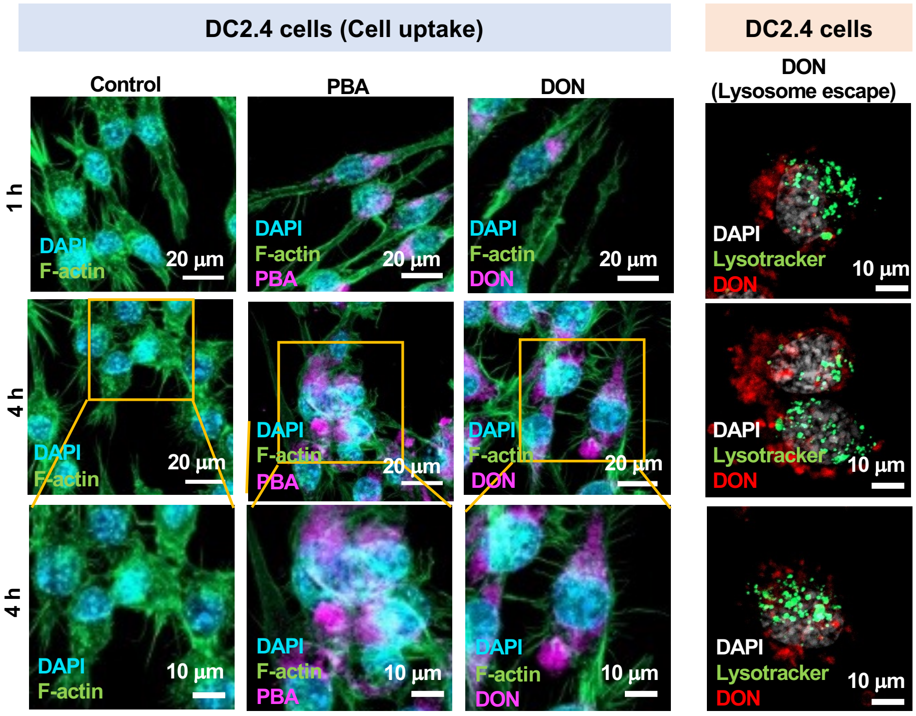

Figure S4. CLSM images of DCs incubated with PBA and DON. Blue, green and purpe represents nucleus stained with DAPI, cytoskeleton with F-Actin, and particles stained with QD, respectively. (i) CLSM images of DCs incubated with DON to evaluate the lysosomal escape effect of DON after HFMF irradiation.

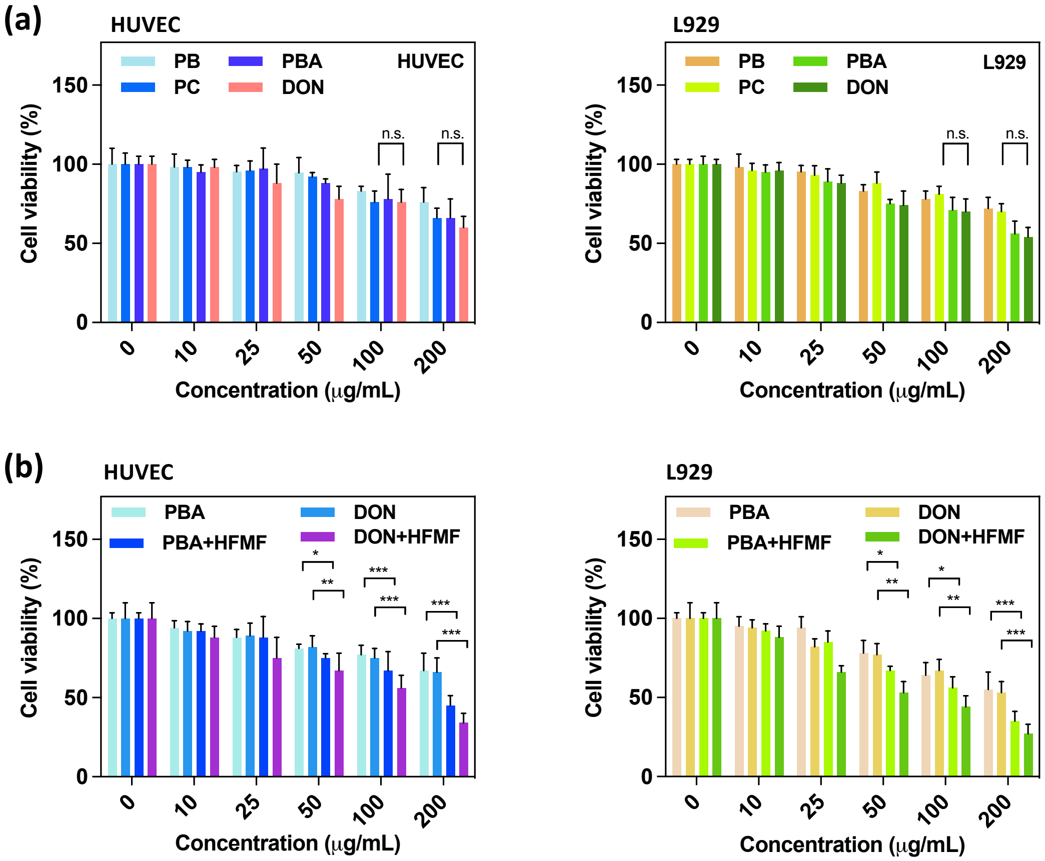

Figure S5. Cell Viability of HUVEC and L929 treated with PB, PC, PBA and DON at various concentrations with and without subjecting to HFMF. Statistical significance was assessed using one-way ANOVA. Data represent mean ± SEM, n = 6. *$p < 0.05$, **$p < 0.01$, ***$p < 0.005$.

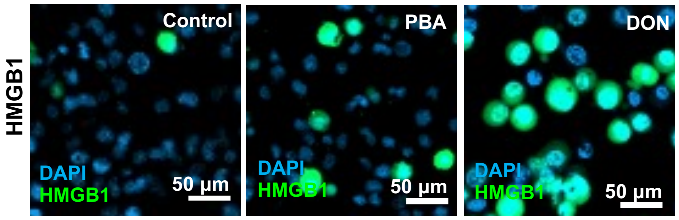

Figure S6. CLSM images of *in vitro* assessments of anti-CRT *via* the catalysis of PBA and DON nanoparticles.

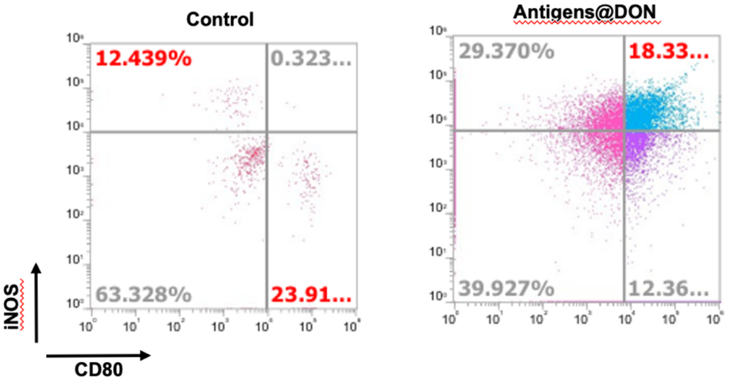

Figure S7. *In vitro* flow cytometry analysis of DCs after post-treatment by antigens@DON.

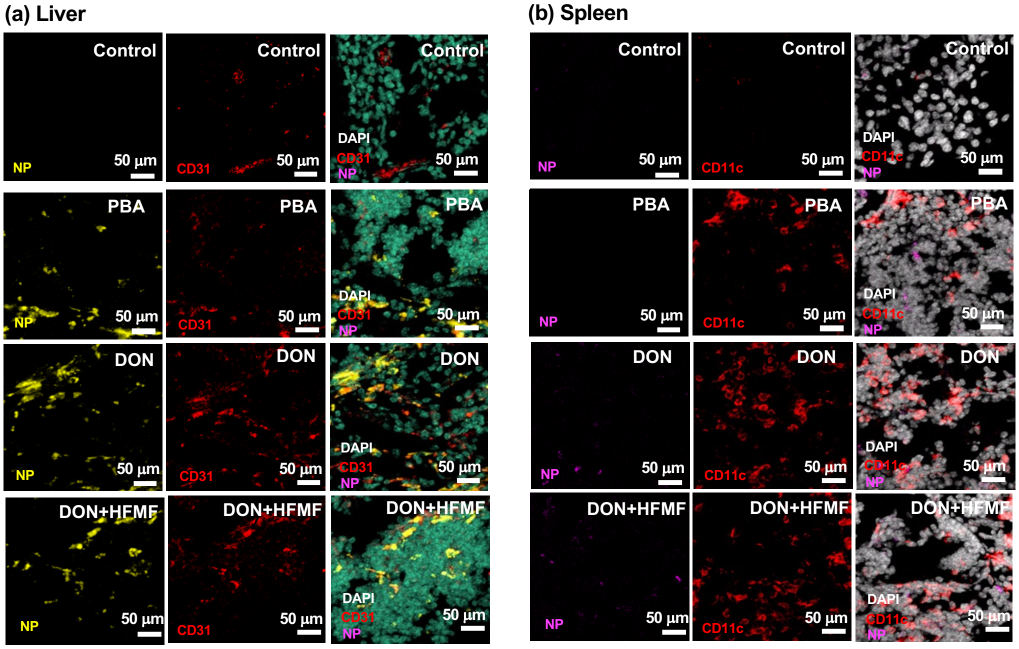

Figure S8. CLSM images of liver and spleen of mice bearing GFP-B16F10 lung metastases after treated with PBA, DON and DON+HFMF after 24 h of treatment, respectively.

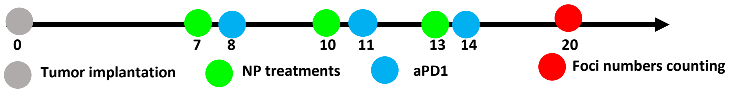

Figure S9. Nanoparticle and anti-PD1 treatment regimens for B16F10 cell metastasis.

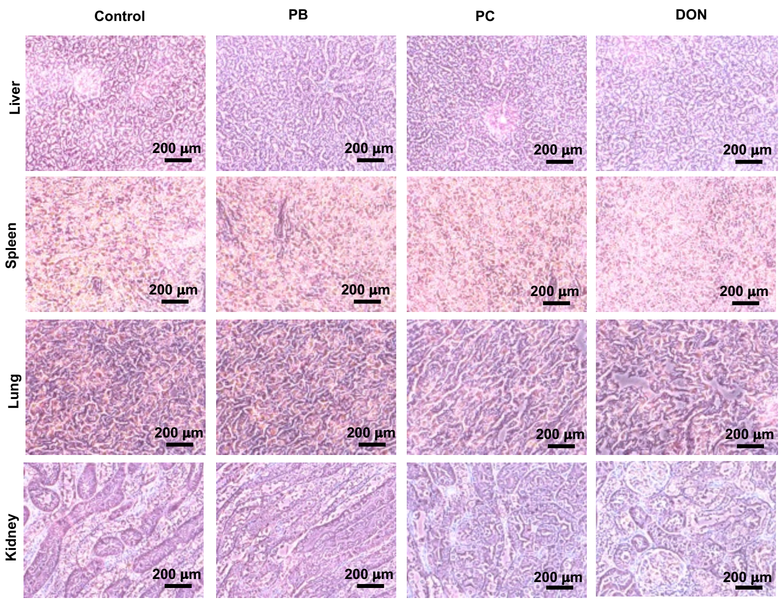

Figure S10. H&E (hematoxylin and eosin) staining of main organs of mice treated with control, PB, PC and DON.

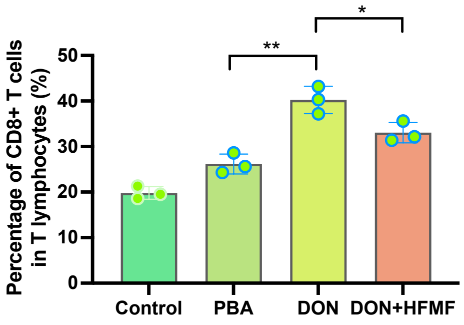

Figure S11. The quantification of $CD8^{+}$ single positive T cells in T lymphocytes. Statistical significance was assessed using one-way ANOVA. Data represent mean ± SEM, n = 6. *$p < 0.05$, **$p < 0.01$.

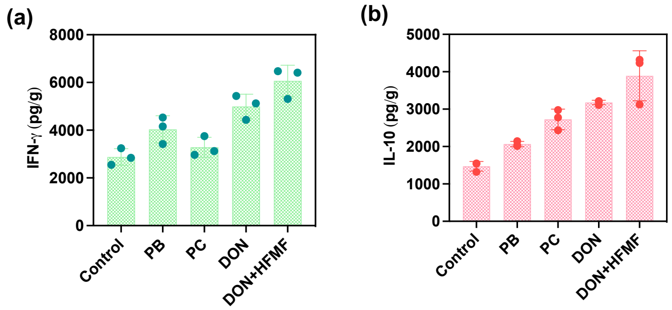

Figure S12. The immune factor concentrations of IFN-γ and IL-10 in lung tissues.

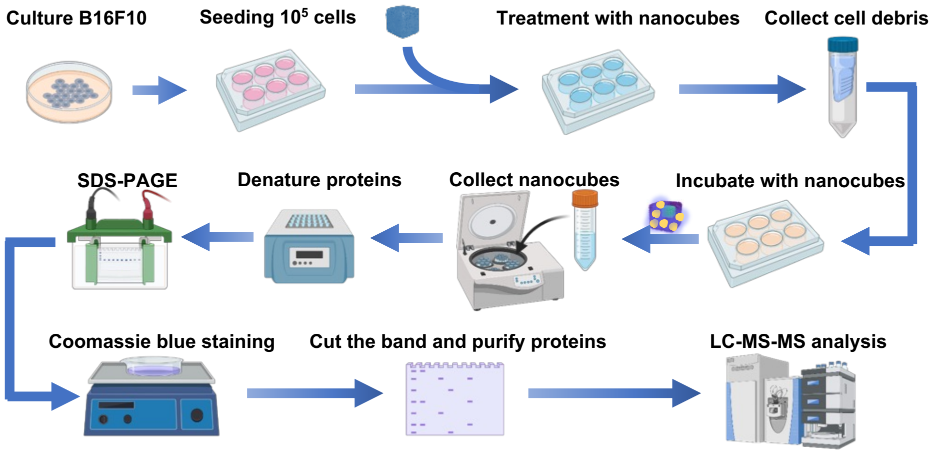

Figure S13. The experimental procedures of analysis of the neoantigens and damage-associated molecular patterns (DAMPs) from B16F10 cells after particles treatment. The released antigen was captured by PBA and DON and analyzed using liquid chromatography mass spectrometry (LC-MS/MS, Orbitrap EliteTM hybrid ion trap-Orbitrap mass spectrometer, ThermoFisher, USA).

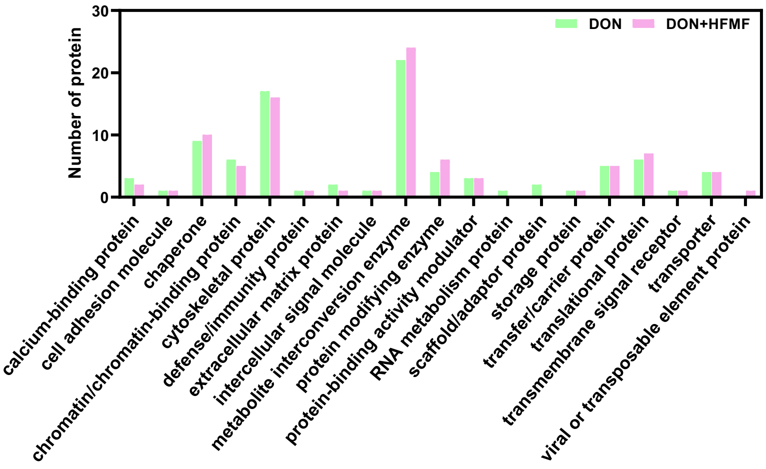

DON		DON+HFMF	
Class	No	Class	No
calcium-binding protein	3	calcium-binding protein	2
cell adhesion molecule	1	cell adhesion molecule	1
chaperone	9	chaperone	10
chromatin/chromatin-binding, or -regulatory protein	6	chromatin/chromatin-binding, or -regulatory protein	5
cytoskeletal protein	17	cytoskeletal protein	16
defense/immunity protein	1	defense/immunity protein	1
extracellular matrix protein	2	extracellular matrix protein	1
intercellular signal molecule	1	intercellular signal molecule	1
metabolite interconversion enzyme	22	metabolite interconversion enzyme	24
protein modifying enzyme	4	protein modifying enzyme	6
protein-binding activity modulator	3	protein-binding activity modulator	3
RNA metabolism protein	1		0
scaffold/adaptor protein	2		0
storage protein	1	storage protein	1
transfer/carrier protein	5	transfer/carrier protein	5
translational protein	6	translational protein	7
transmembrane signal receptor	1	transmembrane signal receptor	1
transporter	4	transporter	4
	0	viral or transposable element protein	1
**Sum**	**89**	**Sum**	**89**

Figure S14. The release of neoantigens and damage-associated molecular patterns (DAMPs) from B16F10 cells by particles treatment. A comprehensive spectrum of related proteins, including 50 highly distinguishable proteins, was observed on PBA and DON.

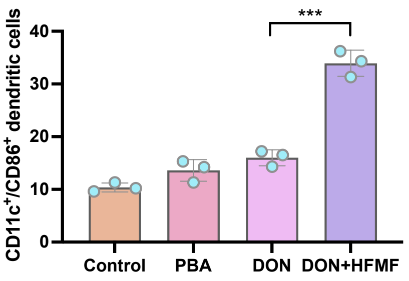

Figure S15. The statistical result for DCs maturation. Statistical significance was assessed using one-way ANOVA. Data represent mean ± SEM, n = 6. ***$p < 0.005$.

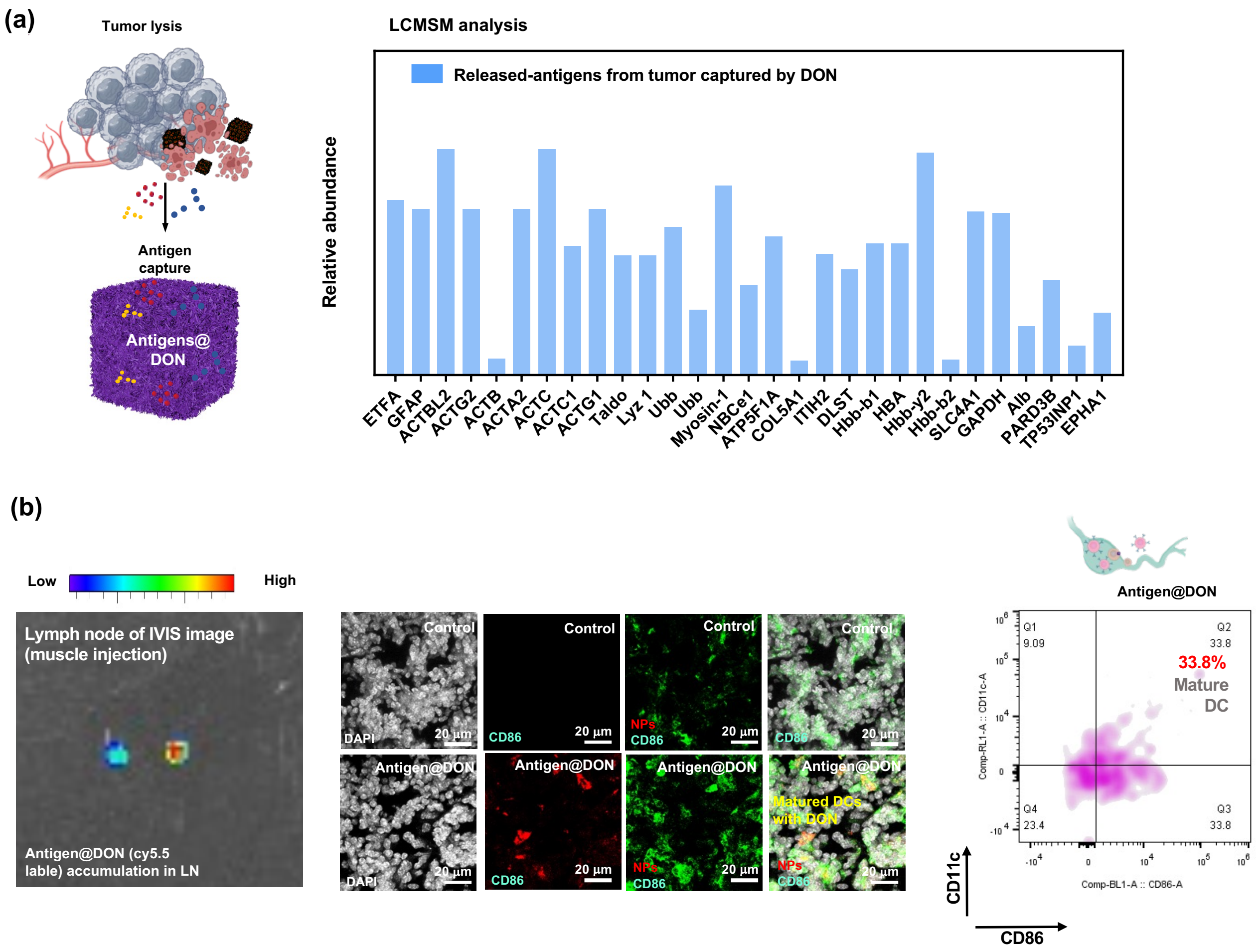

Figure S16. (a) Tumor-released antigens captured by DON. (b) IVIS and CLSM image of dissected lymph node tissue 24 after injection. White, green, and red fluorescence represent nuclei stained with DAPI, DCs labeled with CD86, and nanoparticles labelled with QDs, respectively. *In vivo* flow cytometry analysis of LN tissue dissected after 24 h post-treatment by antigens@DON.
